# Reduction techniques for survival analysis

**DOI:** 10.1007/s10985-026-09714-0

**Published:** 2026-05-10

**Authors:** Johannes Piller, Léa Orsini, Simon Wiegrebe, Sophie Hanna Langbein, Lukas Burk, John Zobolas, Philip Studener, Markus Goeswein, Andreas Bender

**Affiliations:** 1 Statistical Consulting Unit (StaBLab), Department of Statistics, LMU Munich, Ludwigstr. 33, 80539 Munich, Germany; 2 Munich Center for Machine Learning (MCML), LMU Munich, Ludwigstr. 33, 80539 Munich, Germany; 3 Oncostat U1018, Inserm, labeled Ligue Contre le Cancer, University Paris-Saclay, 114 rue Edouard Vaillant, 94800 Villejuif, France; 4 Department of Genetic Epidemiology, University of Regensburg, Franz-Josef-Strauß-Allee 11, 93053 Regensburg, Germany; 5 Leibniz Institute for Prevention Research and Epidemiology - BIPS, Achterstraße 30, 28359 Bremen, Germany; 6 Faculty of Mathematics and Computer Science, University of Bremen, Bibliothekstr. 1, 28359 Bremen, Germany; 7 Machine Learning Consulting Unit (MLCU), Department of Statistics, LMU Munich, Ludwigstr. 33, 80539 Munich, Germany; 8 Department of Cancer Genetics, Institute for Cancer Research, Oslo University Hospital (OUS), Ullernchausseen 64-66, 0379 Oslo, Norway; 9 Department of Biostatistics, Oslo Centre for Biostatistics and Epidemiology (OCBE), University of Oslo (UiO), Ullernchausseen 64-66, 0379 Oslo, Norway; 10 Department of Statistics, LMU Munich, Ludwigstr. 33, 80539 Munich, Germany

**Keywords:** Reduction techniques, Survival analysis, Piecewise exponential, Discrete time survival analysis, Pseudo values

## Abstract

In this work, we discuss what we refer to as reduction techniques for survival analysis, that is, techniques that “reduce” a survival task to a more common regression or classification task, without ignoring the specifics of survival data. Such techniques particularly facilitate machine learning-based survival analysis, as they allow for applying standard tools from machine and deep learning to many survival tasks without requiring custom learners. We provide an overview of different reduction techniques and discuss their respective strengths and weaknesses. We also provide a principled implementation of some of these reductions, such that they are directly available within standard machine learning workflows. We illustrate each reduction using dedicated examples and perform a benchmark analysis that compares their predictive performance to established machine learning methods for survival analysis.

## Introduction

Survival Analysis is an important branch of statistics that deals with time-to-event outcomes. Because of its importance in many diverse areas of application, adaptation of machine and deep learning techniques to survival analysis has received increased interest in recent years.

Generally, we are interested in inference about the outcome of interest (time-to-event) $Y>0$, for example by estimating the distribution of the event times or their expectation. In survival analysis, this is complicated by (right-, left- or interval-) censoring of individual observations as well as (left- or right-) truncation in the data. Additional complications arise when we can observe the same event type multiple times (recurrent events) or when censoring times cannot be assumed to be independent of event times (competing risks).

Several techniques have been developed over the years to deal with such data, prominently the non-parametric Kaplan–Meier (KM; Kaplan and Meier 1958) and Aalen–Johansen (AJ; Aalen and Johansen 1978) estimators, the partial likelihood-based Cox Proportional Hazards (PH) model (Cox 1972, 1975), as well as parametric likelihood-based approaches (Kalbfleisch 2002) and their respective extensions. Since the 2000s, machine learning (ML) approaches have been adapted to survival analysis, including penalized Cox models (Simon et al. 2011; Goeman 2010), variants of random forests (Ishwaran et al. 2008b, 2014; Jaeger et al. 2019), boosting based approaches (Schmid and Hothorn 2008; Binder et al. 2009; Reulen and Kneib 2015), and Bayesian methods (Sparapani et al. 2016; Madjar et al. 2021).

The methodological adaptation of artificial intelligence (AI) methods to survival analysis has been summarized comprehensively in a survey by Wang et al. (2019) for ML methods and a review by Wiegrebe et al. (2024) for Deep Learning (DL) methods. While this illustrates that AI-based survival analysis is a growing field of research, various factors limit its applicability, particularly for practitioners:

- Both Wang et al. (2019) and Wiegrebe et al. (2024) identify only few methods that explicitly go beyond the single-event, right-censored data setting.
- Many research papers only provide insufficient code or proof-of-concept implementations that are hard to use in standard machine learning workflows which require standardized pre-processing, resampling, hyperparameter tuning and evaluation capabilities in addition to the actual learning algorithm.
- There is often a considerable delay between the development and implementation of a method for regression or classification tasks and its adaptation to survival analysis.

**Fig. 1:**
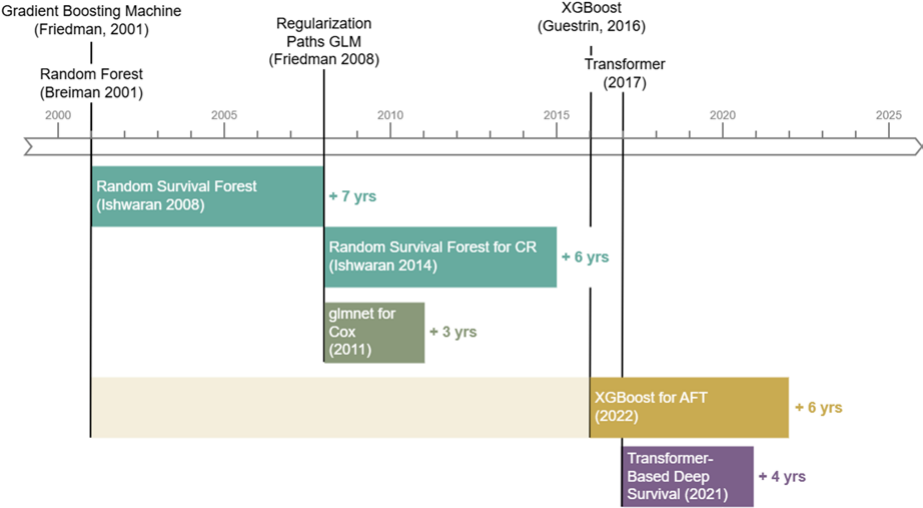
Timeline illustrating the delay between development of new ML methods or software for regression and classification and their adaptation to survival analysis

This is illustrated in Fig. 1. There was a 7-year delay between the proposal of random forests for regression and classification in Breiman (2001) and its adaptation to single-event, right-censored data in Ishwaran et al. (2008a). The extension to competing risks (Ishwaran et al. 2014) took another 6 years. Similar delays can be observed for boosting (Chen and Guestrin 2016a; Barnwal et al. 2022), regularized regression models (Friedman et al. 2010; Simon et al. 2011), and deep learning (e.g., Vaswani et al. 2017; Hu et al. 2021).

Available adaptations in research papers often suffer from point (b), while implementations in popular general purpose software often suffer from point (a). For example, the popular XGBoost library (Chen and Guestrin 2016a) that was used in many winning submissions in Kaggle competitions was extended to fit accelerated failure time models 6 years after the initial publication (although the software had been around for longer), but only learns the scale parameter of the distribution and thus does not provide the full distribution estimation. There is now also an implementation of the Cox model in the XGBoost software, but it natively only returns the risk-score. Both implementations only deal with single-event, right-censored data.

**Fig. 2:**
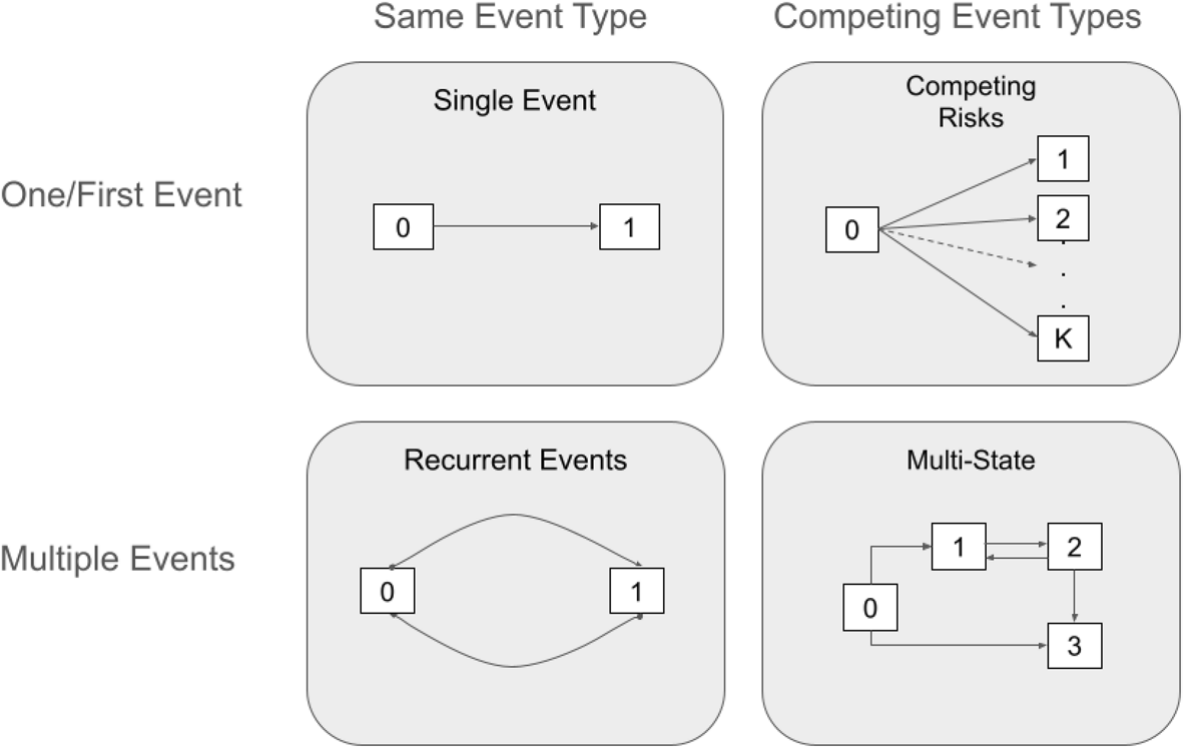
Schematic representation of different survival tasks. Rows indicate if we observe (or are interested in) time to one or first event, or if we are interested in and have observed multiple events. Columns indicate whether we consider occurrences of a single event type or different competing event types

In this work, we consider reduction techniques for survival analysis, that is, techniques that transform a survival task to a more standard regression or classification task. Here we use the term *reduction* in the sense of Beygelzimer et al. (2008, 2016), where “a complex problem is decomposed into simpler subproblems so that a solution to the subproblems gives a solution to the complex problem” (Beygelzimer et al. 2016). This notion differs from *dimensionality reduction* as commonly used in statistics. Rather, it aligns with its use in machine learning, e.g., transforming a multiclass classification problem into multiple one-vs-all tasks. We categorize survival tasks into two dimensions, as these also dictate the pre- and post-processing steps as well as the applicability of the different reduction techniques. The first dimension is the type of censoring or truncation, where we differentiate between left-, right- and interval-censoring, as well as left- and right-truncation (see Wiegrebe et al. 2024 for examples). The second dimension is the number and type of events observed or equivalently the type of transitions we want to model. The latter is illustrated in Fig. 2, where we differentiate between the number of events observed per observation unit (one/first or multiple) and the number of event types observed (one/same or competing event types). Arrows indicate possible transitions between different states, usually starting from an initial state 0. The most general case is the multi-state process (bottom right) with multiple transitions and the possibility of back-transitions (see Sect. 2.2).

Ideally, we want our ML and DL methods to be able to deal with as many of these survival tasks as possible. As mentioned before, however, many published methods for ML-based survival analysis focus on single-event, right-censored data. As we will show later, some reduction techniques cover many—though not all—of these settings, and there are variations depending on the reduction technique of choice. These techniques

- are valid methods for survival analysis that appropriately deal with censoring and/or truncation,
- do not make (strong) assumptions about the underlying distribution of event times,
- are applicable to many survival tasks, including competing risks and multi-state settings,
- can predict different quantities of interest in survival analysis, including (discrete) hazards, survival probabilities, cumulative incidence functions, conditional on features,
- can use any off-the-shelf implementation of ML or DL methods for regression or classification (depending on the reduction technique),
- can (explicitly) model time-varying effects and thus deal with non-proportional hazards,
- and have competitive performance compared to specialized survival learners.

**Fig. 3:**
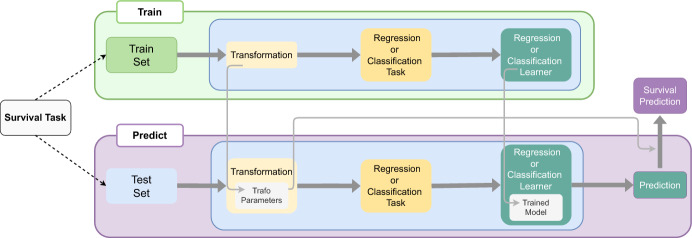
Schematic representation of a reduction ML pipeline. The survival task is split into training and test data. In the training step (top), the survival task is transformed to a regression or classification task, depending on the chosen reduction method and specifics of the underlying survival task. A regression or classification learner is then trained on the transformed data. During the prediction step (bottom), new test data undergo the same transformation using parameters derived during training. Subsequently, the trained model generates predictions in the regression or classification domain, which are then mapped back to survival prediction types according to the reduction strategy and the transformation parameters

The general procedure is visualized in Fig. 3: Reduction techniques for survival analysis transform a survival task to a regression or classification task through a dedicated pre-processing step. While the specifics of the pre-processing depend on the choice of reduction technique and the specific survival task at hand (type of censoring/truncation, presence of competing risks, etc.), upon completion the transformed data can be analyzed using standard methods without further modifications to the learner of choice or the need for survival analysis-specific loss functions. In a predictive modeling context, the transformation parameters derived from the training data are reused to process the test data and the model trained on the transformed training set is then applied to generate predictions, which are subsequently mapped back to survival outputs (survival probabilities, cumulative incidence functions, etc.), if necessary.

The reduction techniques considered in this work are piecewise exponential models (PEM), discrete-time (DT) methods, pseudo values (PV), censoring weighted binary classification (IPCW) and continuous ranking method (CRM), which we group into reductions that estimate the entire event time distribution (PEM, DT) and reductions that are primarily used to obtain a point estimates of a quantity of interest. While the individual techniques that will be discussed in this work have been proposed separately in the literature as stand-alone methods for survival analysis in their own right and partially adapted to the ML setting, to our knowledge they have not been considered jointly as reduction techniques.

The contributions of this work are:

- a unified framework for reduction technique pipelines for survival analysis
- overview of strengths and weaknesses of the different techniques and their applicability to different survival tasks
- a unified, user-friendly implementation of various reduction techniques that can be integrated into a standard machine learning workflows
- quantitative benchmark comparison of the different reductions compared to established statistical machine learning methods for survival analysis

Table 1 provides an overview of the reduction techniques discussed in this paper, along with their respective prediction type, target of estimation, target/label, and survival quantities of interest.

**Table 1:** Overview of five reduction techniques for survival analysis in terms of their prediction type, target of estimation, target/label, prediction task, and survival quantities of interest, with observations *i*, time *t*, discrete time *j* at time *t* and$j_0$at time *s*, transitions *k* with initial state *o* and end state$\ell$

	PEM (Sect. 3.2)	DT (Sect. 3.3)	IPCW (Sect. 4.1)	CRM (Sect. 4.2)	PV (Sect. 4.3)
Prediction type	Distribution	Distribution	Point estimate	Point estimate	Point estimate
Target of estimation	$h(t|\mathbf{x})$ (Eq. 9)	$h(j|\mathbf{x})$ (Eq. 14)	$\pi (\mathbf{x})$ (Eq. 24)	$\rho (\mathbf{x}_i)$ (Eq. 27)	$\theta (t|\mathbf{x})$
Target/label	$d_{ij}$ (Eq. 8)	$d_{ij}$ (Eq. 8)	$e_i$ (Eq. 25)	$\rho _i$ (Eq. 37)	$\theta _i$ (Eq. 38)
Prediction task	Regression	Classification	Classification	Regression	Regression
$\hat{S}(t|\mathbf{x})$	$\exp (-\int _0^t \hat{h}(u|\mathbf{x}) \mathrm{d}u)$ (Eq. 2)	$\prod \nolimits _{l=1}^{j} (1-\hat{h}(l|\mathbf{x}))$ (Eq. 15)	$1-\hat{\pi }(\mathbf{x})$	–	${\widehat{\theta }}_i(t) = n{\widehat{S}(t)}-(n-1){\widehat{S}(t)}^{-i}$ $\widehat{S}(t)$ estimated from the Kaplan–Meier estimator (Eq. 39)
$$\widehat{CIF}_k(t|\mathbf{x})$$	$$\int ^t_0 h_k(u) S(u) \ du$$ (Eq. 6)	$$\sum _{l=1}^j \hat{h}_k(l|\mathbf{x}) \cdot $$$$\hat{S}(l-1|\mathbf{x})$$ (Eq. 22)	–	–	$$\widehat{\theta }_{ki}(t) = n \widehat{CIF_k}(t) - (n - 1) \widehat{CIF_k}^{-i}(t)$$ $$\widehat{CIF_k}(t)$$ estimated from the Aalen–Johansen estimator (Eq. 42)
$$\hat{\mathbf{P}}(s, t)$$	(Eq. 7)	$$\prod \nolimits _{l=j_0}^{\,j}$$$$\left( {\mathbf {I}} + {\mathbf {d}}\hat{{\mathbf {H}}}(l|\mathbf{x})\right) $$ with $$\hat{{\mathbf {H}}}(j|\mathbf{x}):= \left( \hat{h}_{o,\ell }(j|\mathbf{x}) \right) _{o,\ell }$$ and$$\hat{h}_{o,o} = 1 - \sum \limits _{\ell \ne o}\hat{h}_{o,\ell }(j|\mathbf{x})$$	–	–	$$\widehat{\theta }_{ki}(t) = n \widehat{P_k}(t)-(n - 1) \widehat{P_k}^{-i}(t)$$ $$\widehat{P_k}(t)$$ estimated from the Aalen–Johansen estimator (Eq. 41)
RMST	$\int _0^{t} \hat{S}(u|\mathbf{x})\textrm{d}u$ (Eq. 3)	$\sum _{l=1}^{j_{t}} \hat{S}(l|\mathbf{x})$ (Eq. 3)	–	–	${\widehat{\theta }_{i}(t) = n\int _0^{t} {\widehat{S}(u)}du - (n-1)\int _0^{t} {\widehat{S}(u)}^{-i}du}$ $\widehat{S}(t)$ estimated from the Kaplan–Meier estimator (Eq. 40)

In Sect. 2 we introduce notation and definitions relevant for the remainder of this work. Sections 3 and 4 introduce the different reduction techniques, starting with the general definition, followed by details regarding the applicability to different survival tasks as well as limitations, and concluding with a “further reading” section. Sect. 5 discusses software implementations for reduction techniques, particularly in a ML context. In Sect. 6, we illustrate the application of the respective reduction techniques to selected data examples, followed by a quantitative comparison to established statistical and ML methods for survival analysis in Sect. 7.2. Section 8 summarizes results and limitations and provides ideas for future avenues of research.

## Notation and definitions

In the following we introduce common quantities that are usually targets of estimation or prediction in survival analysis. For simplicity, these terms are introduced without dependence on features, but the equations are equally valid in presence of features.

### Quantities of interest

In this section, we briefly recap different functions used to represent (summaries of) the event time distribution, frequently targets of estimation in survival analysis. Let $Y>0$ be the random variable representing event times with realizations *y* and let $t > 0$ be some arbitrary time point. The hazard function, given by

1 $$\begin{aligned} h(t) = \lim _{\varDelta \searrow 0}\frac{P(t < Y \le t + \varDelta |Y \ge t)}{\varDelta }, \end{aligned}$$

is the instantaneous risk to observe an event given that the event has not been observed up until that point. It is a frequent target of estimation, especially among non- and semi-parametric methods such as Cox-type models. Additionally, the hazard rate is often used to construct the survival function via the relationship

2 $$\begin{aligned} S(t) = P(Y > t) = \exp (-H(t)) = \exp \left( -\int _0^t h(u)\ du\right), \end{aligned}$$

where $H_Y(t)$ is the cumulative hazard function.

Finally, we define the restricted mean survival time (RMST) (Irwin 1949; Zhao et al. 2016) as

3 $$\begin{aligned} \mu _{t} = \mathbb {E}(\min (Y, t)) = \int _0^{t} S(u)\textrm{d}u, \end{aligned}$$

which has become popular recently, especially as an intuitive and clinically meaningful measure of feature and treatment effects in randomized clinical trials, in particular when the PH assumption is violated (Royston and Parmar 2011, 2013).

### Beyond single-event setting

As depicted in Fig. 2, a time-to-event process can be represented in terms of transitions between different states. States can be *transient* (transitions to another state possible) or *absorbing* (no further transitions possible, e.g., death).

In the competing risks setting, some states may be transient, but the focus is on the first of multiple mutually exclusive events, without modeling further transitions. Thus, there are *q* distinct competing states, allowing transitions of the form $0 \rightarrow k$, with $k \in \{1, \ldots , q\}$.

Recurrent events describe a setting with repeated occurrences of the same non-terminal event, such as non-fatal respiratory infections. If, in addition, a terminal event is present, it needs to be accounted for, usually by casting it as a multi-state problem.

Single-event, recurrent events, and competing risks settings can be viewed as special cases of the multi-state setting. Let $K \in \{1,\ldots ,q\}$ be a random variable representing a state (competing risks) or transition (e.g., $0\rightarrow 1$, $0\rightarrow 3,\ldots,2\rightarrow 3$ in Fig. 2). Finally, let *k* denote realizations of *K* and let $\mathscr {F}_t$ denote the available information right before time *t*. We extend Eq. (1) to the general multi-state transition rate

4 $$\begin{aligned} h_{k}(t) = \lim _{\varDelta \searrow 0}\frac{P(t \le Y \le t + \varDelta , K = k|Y \ge t, \mathscr {F}_t)}{\varDelta }. \end{aligned}$$

In the competing risks setting, $h_k$ are the cause-specific hazards $h_{0\rightarrow k}$. In the multi-state setting, these are transition-specific hazards $h_{o\rightarrow \ell }(t)$, where *o*, $\ell$ are the initial state and end state, respectively.

The all-cause hazard, which is the total hazard of all possible transitions, is defined as

5 $$\begin{aligned} h(t) = \sum _{k=1}^{q}h_{k}(t). \end{aligned}$$

Once the transition hazards for the different settings have been estimated, further quantities of interest can be obtained from them using closed formulae. In the single-event case, this is typically the survival curve (Eq. (2)). In the competing risks setting, we are often interested in the cumulative incidence function (CIF)

6 $$\begin{aligned} CIF_k(t) = P(Y \le t, K = k) = \int ^t_0 h_k(u) S(u) \ du, \end{aligned}$$

where *S*(*u*) is the all-cause survival probability derived via Eqs. (5) and (2).

In the multi-state setting, we are often interested in the transition probabilities $P_{o\ell }(s, t)$, i.e., the probability to transition from state *o* to state $\ell$ between times *s* and *t*. These can be obtained via the empirical transition matrix (Aalen and Johansen 1978)7where $d\mathbf{H}(t)$ is the matrix of cumulative hazard rate differences $H_{k}(t + \varDelta t) - H_{k}(t)$ (cf. Beyersmann et al. 2012), which can be calculated directly from estimates of Eq. (4).

## Reductions for partition-based hazard estimation

### Partitioning of the follow-up

**Fig. 4:**
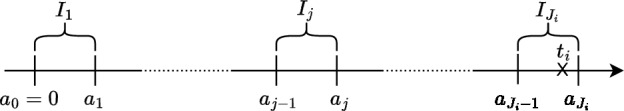
Schematic representation of the partitioning of the continuous follow-up time of subject *i* using cut points $a_j$, $j \in \{1, \ldots , J_i\}$. The corresponding partitioning of the survival dataset is illustrated in Table 2

Some reduction techniques partition the time axis into *J* intervals, estimating a constant or discrete-time hazard within each. As shown in Fig. 4, the *j*th interval is $I_j:= (a_{j-1}, a_j]$, and $J_i \in \{1, \ldots , J\}$ denotes the index corresponding to the interval containing the observed time of subject *i*, $t_i$, with $t_i \in I_{J_i} = (a_{J_i-1}, a_{J_i}]$ and $i \in 1,\ldots ,n$. Intervals may be equidistant or vary in length.

This partitioning is used to create a new long-format dataset with multiple rows per subject, each corresponding to each time interval for which the subject is still (at least partially) at risk (i.e., $\forall j \in \{1, \ldots , J_i\}$). This is done by defining the following variables for each subject interval pair (*i*, *j*) (with corresponding observed time $t_i$, which is the minimum of the subject’s true event time $y_i$ and their censoring time $c_i$):

- the event-specific event indicator
  8 $$\begin{aligned} d_{ij} = {\left\{ \begin{array}{ll} 1 & \text {if } t_i \in I_j \wedge d_i = 1 \\ 0 & \text {else} \end{array}\right. } , \end{aligned}$$
- the time at risk
  $$\begin{aligned} t_{ij} = {\left\{ \begin{array}{ll} a_j - a_{j-1} & \text {if } a_j< t_i\\ t_i - a_{j-1} & \text {if } a_{j-1} < t_i \le a_j\end{array}\right. }, \end{aligned}$$
- and an “offset” to model the rate of events per unit time
  $$\begin{aligned} o_{ij} = \log (t_{ij}), \end{aligned}$$

where $d_i$ is the usual status indicator.

This data transformation is illustrated in Table 2. Left-truncation, time-varying features, and time-varying effects all require similar data transformations and can thus be naturally incorporated. In particular, since $t_{ij}$ becomes just another feature in this transformed data, time-varying effects simply become interaction terms with time. Additional cut points may be necessary, e.g., at the times when time-varying features change.

**Table 2:**
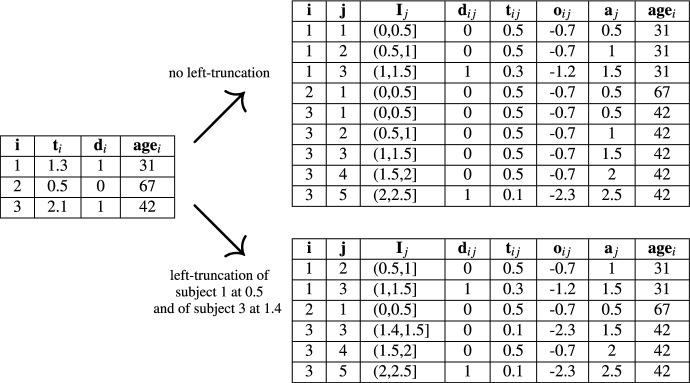
Illustration of survival data transformation due to partitioning of follow-up time

Starting from a standard survival dataset with a single row per subject containing the ID *i*, observed time *t*_*i*_, event indicator *d*_*i*_, and features (here: baseline age), the follow-up time is partitioned into time intervals (here: equidistant with *a*_0_ = 0, *a*_1_ = 0.5, *a*_2_ = 1, etc.) as illustrated in Fig. 4. Correspondingly, the dataset is transformed into an expanded long-format dataset with one row per subject and time interval. The bottom left table additionally incorporates left-truncation ($t_1^L=0.5$ and $t_3^L=1.4$) by removing or restricting intervals prior to the respective subjects’ left-truncation times

For competing risks, multi-state, and recurrent events analyses, the data must be further transformed—or, to be precise, augmented.

In the context of competing risks, a separate dataset for each of the *q* competing events is created, where for cause *k* we replace the interval-specific event indicator $d_{ij}$ by a cause-specific indicator

$$\begin{aligned} d_{ijk} = {\left\{ \begin{array}{ll} 1 &\quad \text {if } d_{ij} = 1 \wedge \ d_{ik} = 1\\ 0 &\quad \text{ else } \end{array}\right. }, \end{aligned}$$

with $d_{ik}$ a binary indicator for whether *i*’s event was of type *k* (or not). In addition, we add a column cause to each of the *q* datasets, taking on the value *k* for all rows of the *k*th dataset. Note that in the *k*th dataset, all events $\tilde{k} \ne k$ are implicitly treated as censoring. This data transformation step can thus also be viewed as augmentation by counterfactual transitions: the row of a subject with event type *k* in the $\tilde{k}$th dataset corresponds to the counterfactual transition from state 0 to state $\tilde{k}$ (and thus $d_{ij\tilde{k}}=0$ even in interval $j_i$).

In multi-state processes, each state and its transitions can be viewed as nested competing risks. Like in competing risks transformations, multi-state data are stacked across all *q* transitions, with an added categorical variable indicating the transition $k \in {0, 1,\ldots, q}$ taken by subject *i*. The binary event indicator $d_{ij}$, defined as in the single-event setting, indicates whether subject *i* experiences the specific transition. Since subjects enter transition-specific risk sets at different times, the transformation includes both the transition time $t_{ij}$ and the state entry time in order to handle the resulting left-truncation. To address censoring from competing events, counterfactual transitions are added as additional rows with the same times, transition identifier, and $d_{ij\tilde{k}}=0$ $\forall \tilde{k} \ne k$.

This follow-up time partitioning and data transformation applies identically to both piecewise exponential and discrete-time reduction techniques (see Sects. 3.2 and 3.3). The key difference is that piecewise exponential models retain exact times at risk $t_{ij}$ (via offsets $o_{ij}$), while discrete-time models ignore exact times at risk, relying solely on the interval index *j*. Importantly, although each subject now has multiple rows in the dataset, which needs to be accounted for during resampling, they can be treated as independent during model training (see details in subsequent sections), such that ML algorithms can be used without modification.

### Piecewise exponential reduction

The piecewise exponential model (PEM) is a reduction technique that transforms a survival task to a Poisson regression task. Initially proposed in the early 1980s (Friedman 1982), it saw limited adoption compared to the Cox model (Cox 1972, 1975), despite their demonstrated equivalence in some situations (Whitehead 1980). However, this model class has regained popularity more recently (see Sect. 3.2.4), with modern versions leveraging efficient algorithms and refined methods for the estimation of the (baseline) hazard and time-varying effects.

#### Single-event setting

Like the Cox model, the PEM estimates the hazard (1) as an exponential function depending on feature vector $\mathbf{x}$, i.e.,

9 $$\begin{aligned} h(t|\mathbf{x}_i) = \exp (g(t, \mathbf{x}_i)), \end{aligned}$$

where $g(t, \mathbf{x})$ is some function of the features and time, learned from the data. Under the PH assumption, we obtain the familiar Cox PH-type hazard $h(t|\mathbf{x}) = h_0(t) \cdot$ $\exp (g(\mathbf{x})) = \exp (\log (h_0(t)) + g(\mathbf{x}))$. In contrast to the Cox model, however, the baseline hazard is estimated jointly with the feature effects.

For the estimation, the data is transformed as illustrated in Table 2 of Sect. 3.1. The newly created status variable $d_{ij}\sim Po(\mu _{ij})$ is then used as outcome in a Poisson model, in which the time at risk $t_{ij}$ enters logarithmically as offset $o_{ij}$, i.e., $o_{ij} = log(t_{ij})$. Intuitively, the model estimates the expected conditional event rate $\mu _{ij}=h_{ij}t_{ij}$ as the product of the hazard rate of subject *i* in the *j*th interval ($h_{ij}$) multiplied with the time subject *i* was at risk for the event ($t_{ij}$).

Using $\eta _{ij} = \log (h_{ij})$, $h(t_i|\mathbf{x}_i)^{d_{i}} = \prod _{j=1}^{J(i)} \exp (d_{ij} \eta _{ij})$, and $S(t_i|\mathbf{x}_i) = \prod _{j=1}^{J(i)}\exp \left( h_{ij}t_{ij}\right)$, we can then express the Poisson log-likelihood as

10 $$\begin{aligned} \ell _{Poi} = \log (L_{Poi})&= \log \left( \prod _{i=1}^n \prod _{j=1}^{J_i} \frac{\mu _{ij}^{d_{ij}}}{d_{ij}!} \exp (-\mu _{ij}) \right) \nonumber \\&= \sum _{i = 1}^{n} \sum _{j=1}^{J_i} \left( d_{ij} \eta _{ij} - \exp (\eta _{ij} + o_{ij}) + d_{ij} o_{ij}) \right) \nonumber \\&\propto \sum _{i = 1}^{n} \sum _{j=1}^{J_i} \left( d_{ij} \eta _{ij} - \exp (\eta _{ij} + o_{ij}) \right) = \prod _{i=1}^n h(t_i|\mathbf{x}_i)^{d_{i}} S(t_i|\mathbf{x}_i). \end{aligned}$$

Thus, the Poisson likelihood is equivalent to the likelihood of the observed survival data, confirming that the reduction to Poisson regression is valid (Holford 1980; Laird and Olivier 1981; Friedman 1982).

For predictions, the offset is ignored, such that the model returns $\hat{h}(t|\mathbf{x})$. Given the piecewise constant nature of the hazard in this model, calculation of the quantities of interest in Eq. (2) becomes straightforward.

#### Multi-state setting

In the multi-state setting, the aim is to estimate transition hazards (see Eq. (4)). Keeping track of different transitions, the data is often given in “start-stop” notation, where each row represents one transition, given by quadruples

$$\begin{aligned} (t^{entry}_{ike}, t^{exit}_{ike}, d_{ike}, \mathbf{x}_{i}), \end{aligned}$$

where $t^{entry}_{ike}$ is the observed entry (i.e., left-truncation) time of subject *i* into the risk set for transition $k=1,\ldots ,q$ in episode $e=1,\ldots ,m$. $t^{exit}_{ike}$ is the respective observed exit time, due to either the transition occurring or censoring. $d_{ike}$ is the corresponding status indicator and $\mathbf{x}_{i}$ the feature row vector.

**Table 3:** Example data (raw, untransformed) for the multi-state model depicted in Fig. 2 in start-stop notation including three subjects, transitions, episodes, and the feature age

i	o	$\ell$	e	t^*entry*^_*ike*_	t^*exit*^_*ike*_	d_*ike*_
1	1	2	1	0	0.5	1
1	2	1	1	0.5	1	1
1	1	2	2	1	3	1
2	0	1	1	0	3	0
3	1	2	1	0	1	1
3	2	3	1	1	2.5	1

The five possible transitions $o \rightarrow \ell$ are $0 \rightarrow 1$, $1 \rightarrow 2$, $2 \rightarrow 1$, $0 \rightarrow 3$, and $2 \rightarrow 3$

With this data structure at hand, we estimate the transition hazard as

11 $$\begin{aligned} h_{ke}(t|\mathbf{x}_i) = \exp (g(t, k, e, \mathbf{x}_i)). \end{aligned}$$

The multi-state framework includes the competing risks setting (dropping *e*) and the recurrent events setting (dropping *k*) as special cases.

The advantage of the PEM approach is that we can use the same estimation in multi-state models as for single-events; only the data transformation differs. Unlike in the single-event transformation, the multi-state version must store information on state entry (because multiple transitions can happen with progressing time) and exit time. It must also include information on possible exit states for each state. Other competing events are considered censoring for the event of interest, i.e., $d_{ike} = 0$, see $i = 3$ in Table 3. Essentially, we either create one dataset for each possible transition (and episode) or include *k*, *e* as features in our hazards. The status indicator is then given as

12 $$\begin{aligned} d_{ijke} = I(d_{ike} = 1 \wedge t_i \in I_j) \in \{0,1\} \sim Po(\mu _{ijke}) \end{aligned}$$

and again used as outcome in a Poisson model, in which the time at risk $t_{ij}$ enters logarithmically as offset $o_{ij}$. Hence, the optimization of the model remains identical to the single-event setting. The hazard can be denoted as

13 $$\begin{aligned} h_{ke}(t|\mathbf{x})&= \exp (\beta _{0ke} + f_{0ke}(t) + g(t, k, e, \mathbf{x})), \end{aligned}$$

where the baseline hazard is given by $h_{0ke}(t) = \exp (\beta _{0ke} + f_{0ke}(t))$, including baseline effects and (non-linear) interactions of transitions *k* and episodes *e* with time *t*. The function $g(t, k, e, \mathbf{x})$ is again some function of the features and time, learned from the data, and can also vary across different transition trajectories and episodes.

Given the piecewise constant nature of the hazard in this model, the calculation of the quantities of interest in (7), especially the construction of $dH_{ke} = H_{ke}(t + \varDelta t | \mathbf{x}) - H_{ke}(t | \mathbf{x})$, becomes straight forward. In the competing risks setting, given the hazard $h_k(t|\mathbf{x})$, the calculation of (6) simplifies.

#### Limitations

In general, PEMs are limited when it comes to handling left- and interval-censored data, but they can accommodate left-truncated and right-censored data through partitioning of the follow-up. This transformation expands the dataset, which can slow down estimation. However, by reducing the problem to a Poisson regression, discretizing follow-up time preserves the essential information while also serving as a tuning parameter that balances speed and precision. Alternatively, techniques for efficient estimation of Poisson regression in the big data context are applicable (Wood et al. 2017; Reulen and Kneib 2015; Sennhenn-Reulen and Kneib 2016). Whenever hazards are not the quantity of interest—e.g., CIF in competing risks settings, transition probabilities in multi-state settings, or RMST for treatment comparison—post-processing the results of PEM-based survival analysis requires some effort. Software packages, e.g., pammtools, already introduce convenience functions for many important quantities.

#### Further reading

Focusing on the limitations of partitioning while still using Poisson Generalized Additive Models to estimate hazards, Argyropoulos and Unruh (2015) presents the Gauss-Labatto quadrature rule (GL) to reduce the needed number of nodes in the partitioned data. Piecewise additive mixed models (PAMMs; Bender et al. 2018) address the arbitrary choice of cut-points by using a fine grid and semiparametric, penalized estimation to model the baseline hazard and time-varying effects, enabling flexible modeling of complex time-dependent features–including weighted cumulative exposures, distributed lag non-linear effects, event-specific hazards, and feature effects. Exploiting this flexibility, Ramjith et al. (2022) discuss the applicability and capability of analyzing recurrent events data with PAMMs, which allows the modeler to include frailties as random effects in the model. The article concludes that PAMMs offer a powerful alternative to Cox-based models, shared frailty models, and variance-adjustment methods for recurrent events, due to their ability to flexibly model complex dependencies and their practical implementation tools. Extending the estimation flexibility following the piecewise exponential reduction, Bender et al. (2021) implemented and evaluated a gradient boosted trees algorithm, using the XGBoost library. The authors show that the boosting approach matches the strong performance of methods like oblique random survival forests and DeepHit across diverse datasets, while requiring less development effort, and note that using sparser cut-points to reduce long-form data size does not impact model performance.

Due to PEM reduction, classical variable selection schemes are available. In more complex settings with a variety of different transitions, e.g., multi-state models, Reulen and Kneib (2015) use boosting to perform a data-driven variable selection. Boosting explores information about conditional transition-type-specific hazard rate functions by estimating the influencing effects of explanatory variables. Alternatively, Sennhenn-Reulen and Kneib (2016) extend the LASSO to structured fusion LASSO tailored for multi-state models. The extension uses *L*1-penalty and a structured fusion approach, with the focus being on relationships between different transitions for multi-state models.

For an illness-death model, a special case of a three-state multi-state model, Cottin et al. (2022) suggest IDnetwork, a deep learning architecture for disease prognostication. Inspired by Lee et al. (2018), the architecture combines a shared subnetwork with three transition-specific subnetworks using multi-task learning with hard parameter sharing to capture common and unique patient patterns. Compared to multi-state Cox models (including spline variants), IDnetwork improves discrimination and calibration, particularly with non-linear data patterns in simulated and real-world cancer datasets.

### Discrete-time reduction

Discrete-time (DT) methods are particularly useful when event times are *intrinsically* discrete (Tutz et al. 2016). However, here we consider them as an approximation for continuous time distributions. To this end, the time-axis can be partitioned into non-overlapping time intervals (as for PEMs, see Sect. 3.1). Analogously to PEMs reducing survival tasks to Poisson regression tasks, discrete-time models reduce survival tasks to binary classification tasks and are therefore very popular among DL-based survival analysis methods (Wiegrebe et al. 2024).

Throughout this section, let $\tilde{Y} \in \{1,\ldots ,J\}$ be the random variable denoting discrete or discretized time. In the latter case, we have $P(Y \in I_j) \Leftrightarrow P(\tilde{Y} = j)$.

The DT hazard

14 $$\begin{aligned} h(j|\mathbf{x}) = P(\tilde{Y} = j | \tilde{Y} \ge j, \mathbf{x}) \end{aligned}$$

represents the probability of the event occurring during the *j*th time interval, conditional on surviving up until the beginning of that interval (see Tutz et al. 2016 for details).

As for the PEM, once the hazard is estimated by a binary classification model (which returns probabilities), other quantities can be directly calculated from the hazard.

For example, the DT survival function is given by

15 $$\begin{aligned} S(j|\mathbf{x}) := P(\tilde{Y}>j|\mathbf{x}) = P(Y > a_j|\mathbf{x}) = \prod _{l=1}^j (1-h(l|\mathbf{x})). \end{aligned}$$

The unconditional probability of the event occurring at time *j* is

16 $$\begin{aligned} P(\tilde{Y} = j|\mathbf{x}) = h(j|\mathbf{x}) S(j-1|\mathbf{x}). \end{aligned}$$

Ignoring the exact event time within an interval thus gives likelihood contributions $P(\tilde{Y}=j|\mathbf{x})$ for subjects that experience an event in interval *j* and $P(\tilde{Y} > j|\mathbf{x})$ for subjects censored in interval *j*. With this, and using the binary event indicators $d_{ij}$ from Sect. 3.1, the likelihood for the observed data can thus be written as (cf. Tutz et al. 2016):

17 $$\begin{aligned} L&\propto \prod _{i=1}^n P(\tilde{Y}=J_i|\mathbf{x}_i)^{d_i} P(\tilde{Y} > J_i|\mathbf{x}_i)^{1-d_i} \nonumber \\&= \prod _{i=1}^n \prod _{j=1}^{J_i} h(j|\mathbf{x}_i)^{d_{ij}} (1-h(j|\mathbf{x}_i))^{1-d_{ij}}. \end{aligned}$$

This likelihood, however, is equivalent to the likelihood of binary responses $d_{ij}$ (with $i=1, \ldots , n$ and $j=1, \ldots , J_i$) from a binary response model $g(P(d_{ij}=1|\mathbf{x}_i))=f(\mathbf{x}_{i})$ with link function *g*(), some predictor function *f*() and $d_{ij} {\mathop {\sim }\limits ^{iid}} Ber(h(j|\mathbf{x}_i))$.

#### Single-event setting

In the single-event setting, any classification algorithm that returns (calibrated) probabilities can be applied to the transformed data. DT survival approaches typically parametrize the discrete hazard function. Thus a simple DT model is the *logistic model* (or *continuation ratio model*), a standard logistic regression model (i.e., using a logit link) for $h(j|\mathbf{x}_i)$, the conditional probability of the event taking place in *j*:

18 $$\begin{aligned} h(j|\mathbf{x}_i)=P\left( d_{ij}=1 | \mathbf{x}_{i}\right) =\frac{\exp \left( \eta _{ij}\right) }{1+\exp \left( \eta _{ij}\right) }, \end{aligned}$$

with linear predictor $\eta _{ij} = \beta _{0j}+\mathbf{x}_{i}^{\top } \boldsymbol{\beta }$ and discrete baseline hazard $\beta _{0j}$.

However, $h(j|\mathbf{x}_i)$ can also be estimated using random forests or gradient boosting for classification (see Sects. 6 and 7.2 for application examples). Section 3.3.4 provides an overview of specific DT methods proposed in the literature.

#### Competing risks setting

We now consider the competing risk setting introduced in Sect. 2.2. In the discrete-time context, the cause-specific hazard is defined as

19 $$\begin{aligned} h_k(j|\mathbf{x}) := P(\tilde{Y} = j, K = k | \tilde{Y} \ge j, \mathbf{x}), \end{aligned}$$

the all-cause hazard as

20 $$\begin{aligned} h(j|\mathbf{x}) := \sum _{k=1}^q h_k(j|\mathbf{x}) = P(\tilde{Y} = j | \tilde{Y} \ge j, \mathbf{x}), \end{aligned}$$

and the all-cause survival function as

21 $$\begin{aligned} S(j|\mathbf{x}) = P(\tilde{Y} > j|\mathbf{x}) = \prod _{l = 1}^{j} (1 - h(l|\mathbf{x})). \end{aligned}$$

From this, the CIF can be derived as

22 $$\begin{aligned} CIF_k(j|\mathbf{x}) = \sum _{l=1}^j P(\tilde{Y} = l, K = k|\mathbf{x}) = \sum _{l=1}^j h_k(l|\mathbf{x}) S(l-1|\mathbf{x}). \end{aligned}$$

The data transformation necessary for competing risks analysis is as described in Sect. 3.1. For estimation of cause-specific hazards, binary classification algorithms can now simply be applied to each cause-specific dataset separately; potentially shared effects across causes can be estimated by instead stacking the *q* datasets and using *k* as a feature. The latter approach is preferred in the machine learning context, as it only requires estimation of one model and shared and cause-specific effects can be learned via interactions between feature *k* and other features (see Sect. 6 for an example). A widely used DT competing risks model is the *multinomial logit model* (Tutz 2011; Agresti 2012), where the cause-specific hazard is defined as

23 $$\begin{aligned} h_k(j|\mathbf{x}) = \frac{\exp (\eta _{kj})}{1 + \sum _{k = 1}^q\exp (\eta _{kj})}. \end{aligned}$$

This is a straightforward generalization of the *logistic model* with the cause-specific linear predictor for event *k* now defined as $\eta _{kj} = \beta _{0kj}+\mathbf{x}^{\top } \boldsymbol{\beta }_k$. This model can further be extended to a multi-state model, by modeling all possible transitions instead of only those originating from state 0 (for details, see Steele et al. 2004; Tutz 2016). While the multinomial logit model has the advantage of not requiring cause-specific dataset transformations, a crucial disadvantage of this approach is that it does not lend itself to *binary* classifiers anymore.

In general, just as any binary classification algorithm can be used for estimation of $h(j|\mathbf{x})$ in the single-event setting, any multiclass classification algorithm (including one vs. all reductions) can be used for estimation of $h_k(j|\mathbf{x})$ in competing risks scenarios.

#### Limitations

DT survival methods are particularly useful in case of interval-censored survival data (e.g., caused by coarse data collection) because of their intrinsic handling of interval-censoring—provided that the intervals are identical across all subjects. We note that the choice of time intervals in DT models has a substantial impact on predictive accuracy, as discretization introduces a trade-off between model flexibility and information loss, making the number of bins a critical tuning hyperparameter (Sloma et al. 2021). However, this also depends on the choice of learner, as some methods have implicit or explicit regularization. Individual-specific interval-censoring renders the individual-specific likelihood contributions much more complex and thus standard binary classification algorithms cannot be applied anymore (Tutz et al. 2016); this also holds for the case of left-censored data. Whenever time is intrinsically continuous, however, discretization via partitioning of follow-up time causes loss of information because only time intervals as a whole are considered whereas information about the exact event time within the intervals—as well as information about interval length—is disregarded (as opposed to PEMs). In addition, as for the piecewise-exponential reduction technique, the partitioning-based data transformation of the discrete-time reduction technique can substantially increase dataset size and predictions of quantities of interest other than the discrete hazard require additional post-processing.

#### Further reading

DT survival analysis originated as a grouped-data version of the Cox PH model with complementary log-log link (*Gompertz model;* Kalbfleisch and Prentice 1973), particularly useful in case of many ties as these do not pose a problem with discretized data. Other statistical discrete hazard models are the *Probit model*, the *Gumbel model*, and the *exponential model* (see Tutz et al. 2016). Survival stacking (Craig et al. 2021) is an alternative approach to reducing survival analysis tasks to binary classification tasks, also via data transformation (“stacking”). Instead of explicitly discretizing follow-up time, survival stacking uses Cox-like risk sets to evaluate likelihood contributions at event time points only. Another popular DT reduction technique is *Multi-Task Logistic Regression* (*MTLR* Yu et al. 2011). The data transformation (including partitioning of follow-up time) is identical to the one presented in Sect. 3.1, except that *MTLR* also assigns entries to a non-censored individual *i* after event occurrence. *MTLR* models interval-specific event indicators jointly to enforce logical consistency across time, requiring a generalized logistic regression and limiting compatibility with standard binary classifiers. It closely resembles the DT logistic model (Eq. 18) when feature effects are allowed to vary over time, effectively estimating time-varying effects. Due to the simplicity of binary classification, discretizing survival data is hugely popular in deep learning (Wiegrebe et al. 2024). While the “natural” loss for DT survival tasks is the negative log-likelihood (see Tutz et al. 2016; Zadeh and Schmid 2020; Wiegrebe et al. 2024), the cross-entropy loss is also popular among DL-based approaches (see, e.g., Ren et al. 2019; Huang et al. 2023)—even though it has been shown to cause biased predictions, poor calibration, and large prediction error due to not fully exploiting the information from uncensored individuals (Zadeh and Schmid 2020).

While we only considered DT approaches parametrizing the discrete hazards, others directly parametrize the probability mass function, such as *TransformerJM* (Lin and Luo 2022) or the popular competing risks method *DeepHit* (Lee et al. 2018).

### Handling of different feature and event modalities

Due to the long format of the transformed data, time-varying features and effects can easily be incorporated into both DT and PEM survival models; for instance, **age**$_i$ (baseline age) in Table 2 can simply be replaced by **age**$_{ij}$ (time-varying age) for each individual *i* and time interval *j*.

Moreover, owing to the simplicity of the binary or Poisson response models fitted to the transformed data, more complex event modalities can easily be modeled: for instance, frailties and recurrent events (through the inclusion of random effects into the linear predictor) or competing risks and multi-state settings (through the inclusion of time-varying effects for different causes/transitions).

Importantly, $h_j(\mathbf{x})$ can represent any function of the feature vector $\mathbf{x}_{i}$, including high-order interactions. This enables direct modeling of complex, non-proportional hazards and time-varying effects without relying on proportionality assumptions. Together with the above-mentioned reduction-based flexibilities, these properties make the PEM and DT approaches well-suited for machine learning algorithms.

## Reductions for quantity estimation at selected time points

### Inverse probability of censoring weighting reduction

The inverse probability of censoring weighting (IPCW) technique handles right-censored survival data by transforming the survival task into a weighted classification problem (Vock et al. 2016). Specifically, IPCW allows models to predict the probability of an event (e.g., an adverse health outcome) occurring before a specific cutoff time or time horizon. Thus, the model predicts a continuous risk score, without estimating the entire survival distribution.

#### Single-event setting

In the IPCW reduction, the prediction target is the conditional probability that the event occurs before or at a fixed cutoff time *t*, given the subject’s features $\mathbf{x}_i$:

24 $$\begin{aligned} \pi (\mathbf{x}_i) := P(y_i \le t \mid \mathbf{x}_i). \end{aligned}$$

The binary event label used for classification is defined as

25 $$\begin{aligned} e_i := {\mathbb{1}}(t_i \le t \text { and } d_i = 1). \end{aligned}$$

The IPCW procedure consists of three steps:

1. Estimate the censoring survival function $\hat{G}(t)$ using the Kaplan–Meier estimator on the training data.
2. Compute observation weights as
  26 $$\begin{aligned} \omega _i := \frac{1}{\hat{G}(\min (t_i, t))}, \end{aligned}$$
  assigning $\omega _i = 0$ to subjects censored before *t*, as their status $e_i$ from Eq. 25 is unknown.
3. Train a binary classification algorithm using the labels $e_i$ and weights $\omega _i$ for the observations. This up-weights individuals with complete follow-up and down-weights those censored before *t*.

The resulting model predictions $\hat{\pi }(\mathbf{x}_i)$ represent a continuous risk score: higher values indicate greater likelihood of experiencing the event before *t*. These scores can also be interpreted as time-specific survival estimates via $S_i(t \mid \mathbf{x}_i) = 1 - \hat{\pi }(\mathbf{x}_i)$.

#### Limitations

IPCW can be incorporated into many standard classification models, provided that they support observation weights (e.g., classification trees, logistic regression). Its application is less straightforward for models like neural networks, where weighting mechanisms are not inherently supported. Moreover, the statistical validity of the method rests on the assumption that the censoring time is independent of both the event time and features. When this assumption is violated, using unconditional Kaplan–Meier weights can potentially lead to biased predictions (Gerds and Schumacher 2006). Instead, more suitable approaches should estimate $\hat{G}(t|\mathbf{x})$ conditionally, using models like the Cox PH model (Cox 1972) or flexible learners such as random survival forests (Ishwaran et al. 2008b), which can better account for feature-dependent censoring.

#### Further reading

Recent IPCW extensions enable handling of competing risks and dependent censoring by integrating IPCW into the resampling step rather than the learning algorithm itself, allowing the use of any standard machine learning method without modification (Gonzalez Ginestet et al. 2021). These advances generalize previous work and improve robustness in complex survival settings.

### Complete ranking method reduction

The Complete Ranking Method (CRM) addresses the challenge of handling right-censored survival data by transforming the survival task into a regression problem through a uniform ranking scheme (Guan et al. 2021). This approach is highly flexible, allowing the use of any regression algorithm, including advanced methods such as neural networks, support vector machines, and gradient boosting trees, making it broadly applicable across various domains—including survival tasks with continuous spatial or temporal data.

#### Single-event setting

In the CRM reduction, the estimation target reflects how likely it is that a given individual *i* experiences the event of interest before another randomly selected subject *j*, i.e., whether $y_i < y_j$. This can be expressed as the expected pairwise comparison outcome:

27 $$\begin{aligned} \rho (\mathbf{x}_i) := \mathbb {E}_j\!\left[ \mathbb {I}(y_i < y_j) \mid \mathbf{x}_i \right] , \end{aligned}$$

where the expectation is taken over a randomly drawn subject *j*.

Since the true event times are not fully observed due to censoring, the indicator $\mathbb {I}(y_i < y_j)$ cannot be evaluated directly. CRM therefore replaces this unobservable quantity with a pairwise probability estimate based on the observed outcomes. For each pair, a relative risk score $r_{ij}$ is computed, representing the conditional probability that individual *i* fails before individual *j*, given their observed outcome times $(t_i, t_j)$ and event indicators $(d_i, d_j)$.

Formally, we define

28 $$\begin{aligned} r_{ij} := P(y_i < y_j \mid (t_i,d_i),(t_j,d_j)). \end{aligned}$$

By construction, $r_{ij} + r_{ji} = 1$, so that each pairwise comparison induces a probabilistic ranking between the two individuals.

The value of $r_{ij}$ depends on the ordering of the observed times $(t_i, t_j)$ and the censoring indicators $(d_i, d_j)$. In total, six distinct cases arise. When the ordering is identifiable from the observed data, $r_{ij}$ is deterministic; otherwise, uncertainty due to censoring is resolved using the Kaplan–Meier estimator.

Let $\hat{S}(t)$ denote the Kaplan–Meier estimate of the survival function. Without loss of generality, we consider the case $t_i < t_j$:

**Case 1:** $d_i = 1, \ d_j = 0$ (event vs. censored)

29 $$\begin{aligned} r_{ij} = 1, \quad r_{ji} = 0. \end{aligned}$$

**Case 2:** $d_i = 1, \ d_j = 1$ (both events)

30 $$\begin{aligned} r_{ij} = 1, \quad r_{ji} = 0. \end{aligned}$$

**Case 3:** $d_i = 0, \ d_j = 1$ (censored vs. event)

31 $$\begin{aligned} r_{ij} = P^*, \quad r_{ji} = 1 - P^*. \end{aligned}$$

where $P^*$ represents the conditional probability that a subject fails in the interval $(t_i, t_j]$, given survival up to time $t_i$, as estimated by the Kaplan–Meier survival function:

32 $$\begin{aligned} P^* = \frac{\hat{S}(t_i) - \hat{S}(t_j)}{\hat{S}(t_i)}. \end{aligned}$$

**Case 4:** $d_i = 0, \ d_j = 0$ (both censored)

33 $$\begin{aligned} r_{ij}&= P^* + \tfrac{1}{2}(1 - P^*), \end{aligned}$$

34 $$\begin{aligned} r_{ji}&= \tfrac{1}{2}(1 - P^*), \end{aligned}$$

In the case of ties in the observed times, i.e. $t_i = t_j$, we distinguish two additional cases:

**Case 5:** $$d_i = d_j$$

35 $$\begin{aligned} r_{ij} = r_{ji} = \tfrac{1}{2}. \end{aligned}$$

**Case 6:** $$d_i \ne d_j$$

36 $$\begin{aligned} r_{ij} = {\left\{ \begin{array}{ll} 1, & \text {if } d_i = 1, \ d_j = 0, \\ 0, & \text {if } d_i = 0, \ d_j = 1, \end{array}\right. } \quad r_{ji} = 1 - r_{ij}. \end{aligned}$$

For each observation *i*, the regression target is obtained by averaging these pairwise scores over all comparison subjects:

37 $$\begin{aligned} \rho _i = \frac{1}{n - 1} \sum _{j \ne i} r_{ij}. \end{aligned}$$

This averaging step yields an empirical estimate of the target quantity in (27), where the unobservable indicators $\mathbb {I}(y_i < y_j)$ are replaced by their pairwise probability estimates $r_{ij}$. In this sense, $\rho _i$ can be interpreted as the estimated probability that subject *i* fails before a randomly selected comparison subject from the dataset. The resulting targets satisfy $\rho _i \in [0,1]$, where larger values indicate higher relative risk compared to other individuals.

A regression model is trained to predict these targets from features, producing continuous predictions $\hat{\rho }(\mathbf{x}_i)$ for new observations.

#### Limitations

Similar to the IPCW technique utilized in Vock et al. (2016), CRM does not directly predict the time to an event; instead, it provides a relative risk ranking of samples. As such, its predictions focus on the likelihood of an individual failing before others rather than estimating the entire survival distribution for a subject. Additionally, CRM has yet to be extended to handle more complex survival scenarios, such as competing risks.

### Pseudo-value reduction

Pseudo-value (PV) regression is a general method to fit a regression model when a suitable nonparametric estimator of the quantity of interest is available (Andersen et al. 2003). Suppose $\widehat{\theta }$ is an unbiased estimator of a quantity of interest $\theta = \mathbb {E}(h(Y))$, where *h* is a known function. We denote the conditional expectation by $\theta _i = \mathbb {E}(h(Y_i)| \mathbf{x}_i)$. For individual *i*, the PV is defined as

38 $$\begin{aligned} \widehat{\theta }_{i} = n\widehat{\theta } - (n-1)\widehat{\theta }^{-i}, \end{aligned}$$

where $\widehat{\theta }^{-i}$ is the value of the estimator when the *i*th individual is removed from the dataset. PVs can be interpreted as an individual contribution to the overall estimate of the quantity of interest.

Notably, in order to use PVs for predicting the different quantities of interest (cf. Sect. 2) based on individual features, we only need to replace $\hat{\theta }$ and $\hat{\theta }^{-i}$ by the respective (non-parametric) unbiased summary statistic based on the whole sample, e.g., Kaplan–Meier estimator for survival probability or Aalen–Johansen estimator for cumulative incidence function and transition probabilities.

The main advantage of using PVs is that they can be computed for each individual at any time, regardless of censoring. Hence, by transforming survival data into PVs, methods usually restricted to uncensored data can be applied. For this reason, PVs are increasingly used in machine learning applications, as they can be treated like any standard outcome variable, enabling the direct use of learning algorithms without requiring adaptations for censored data (Rahman et al. 2021; Bouaziz 2023; Cwiling et al. 2023). However, it is important to note that the PV is an asymptotically valid approximation of the quantity of interest (Overgaard et al. 2017).

Once calculated, PVs can be used as an outcome variable in a regression setting. In a statistical modeling context, generalized linear models are used to relate the quantity of interest to features, often estimated via generalized estimating equations (GEE; Liang and Zeger 1986). The choice of link function and variance structure then dictates the interpretation of estimated coefficients and validity of inference. For an overview of PV approaches in a statistical setting, see Andersen and Pohar Perme (2010).

**Fig. 5:**
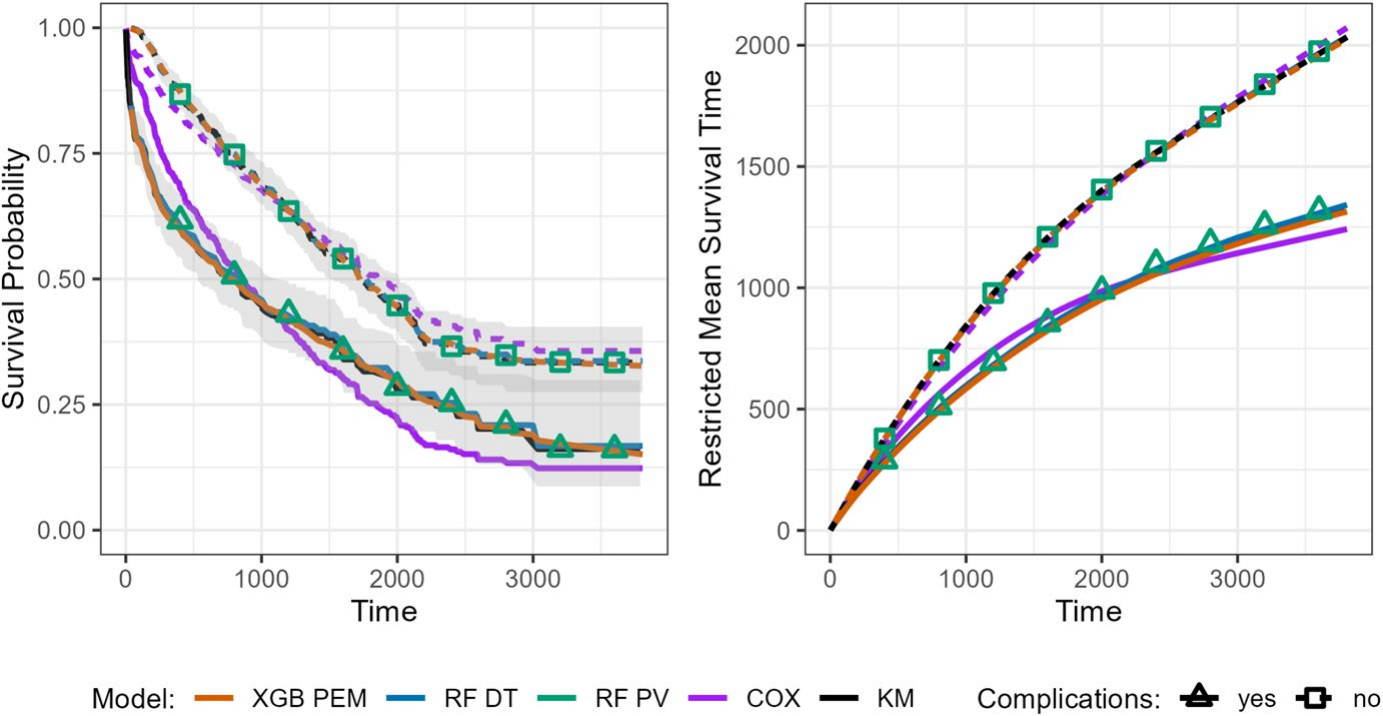
Comparison of survival probabilities and RMST across reduction techniques for the tumor dataset. This figure shows predicted survival probabilities (upper panel) and RMST (lower panel) stratified by complication status, for the three reduction techniques PEM, DT, and PV, as well as for the non-parametric Kaplan–Meier (KM) estimator and Cox PH. For PEM, XGBoost was employed; for DT and PV, random forests were applied. For the DT and PEM approaches and the Kaplan–Meier estimator, curves of the survival function and RMST are shown; for the PV approach, point estimates at specific time points are displayed for both survival probabilities and RMST

**Fig. 6:**
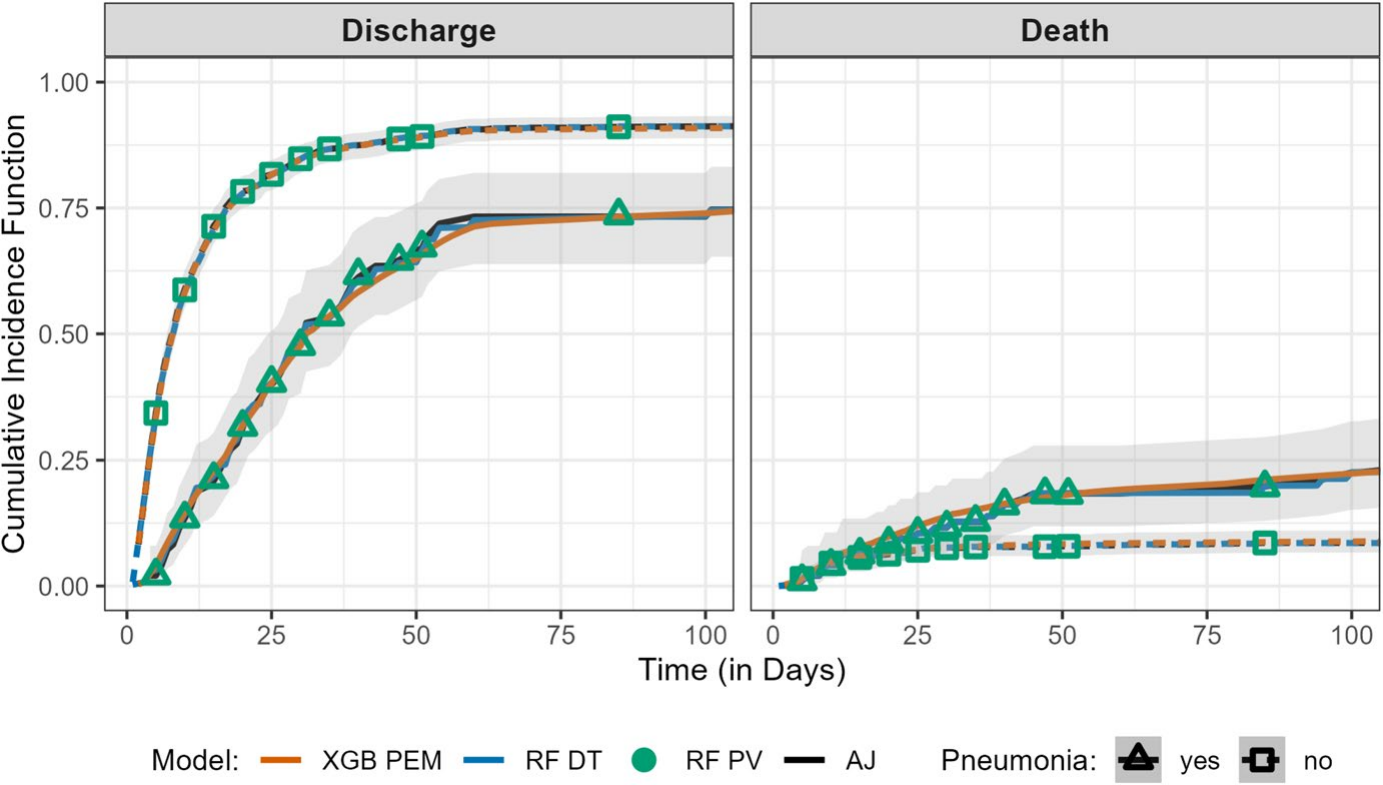
Comparison of cumulative incidence across reduction techniques for the competing risks sir.adm dataset. This figure shows predicted survival probabilities by event type and stratified by complication status, for the three reduction techniques DT, PEM, and PV as well as for the non-parametric Aalen–Johansen (AJ) estimator. For PEM, XGBoost (XGB PEM) was employed; for DT and PV, random forests (RFC DT/PV) were applied. For the DT and PEM approaches and the Aalen–Johansen estimator, curves of the survival function are shown, whereas for the PV approach, point estimates of the survival probability at specific time points are shown

In most cases, PV approaches are not used to improve estimations compared to standard survival methods, but rather to provide an easier alternative in case standard survival methods involve complex modeling. Here we consider PVs in a predictive modeling context, where machine learning models for regression can be used directly to predict the quantity of interest conditional on features (see Figs. 5 and 6 in Sect. 6).

#### Survival probability estimation

Using the Kaplan–Meier estimator, we can compute PVs based on the estimated survival probability. Let *t* be a specific time of interest. The PVs are defined as

39 $$\begin{aligned} {\widehat{\theta }}_i(t) = n{\widehat{S}(t)} - (n-1){\widehat{S}(t)}^{-i}, \end{aligned}$$

where ${\widehat{S}(t)}$ is the Kaplan–Meier estimate of the survival probability at time *t* and ${\widehat{S}^{-i}(t)}$ is the Kaplan–Meier estimator of the survival probability at time *t* after removing the *i*th individual from the dataset.

In order to obtain survival probability estimates at different time-points ${t_1,\ldots , t_K}$, PVs are calculated at each time-point and a regression algorithm is fit to each data set. Alternatively, the data sets can be stacked and estimated jointly. In the ML context, estimation at different time points can be achieved by stacking the PVs for each time-point and adding $t_k, k =1,\ldots ,K$ as an additional feature.

#### Restricted mean survival time

PVs are particularly useful in adjusting the estimation of restricted mean survival time (RMST) conditional on features. Classical survival models estimate the restricted mean survival time by first modeling the survival function and then integrating it between 0 and *t* (Karrison 1987; Zucker 1998). This approach is often computationally expensive and only gives an indirect interpretation of feature effects. On the other hand, PVs offer a direct way of regressing the RMST on features. For this application, one PV is defined for each subject as

40 $$\begin{aligned} {\widehat{\theta }_{i}(t) = n\int _0^{t} {\widehat{S}(u)}du - (n-1)\int _0^{t} {\widehat{S}(u)}^{-i}du}. \end{aligned}$$

The conditional RMST can then be estimated as

41 $$\begin{aligned} \mathbb {E}(\min (Y_i, t)| \mathbf{x}_i)) = f(\mathbf{x}_i), \end{aligned}$$

where $f(\mathbf{x}_i)$ is a function of the features learned by the regression algorithm of choice, e.g., deep neural networks (Zhao 2021), Super Learners (Cwiling et al. 2024) or random forest (Schenk et al. 2025). See also Sect. 6 for an illustration using random forests.

#### Multi-state setting

The PV approach, originally developed for modeling state probabilities in multi-state models, is broadly applicable, i.a. to the illness-death model (Andersen et al. 2003) or more complex multi-state models (Andersen and Klein 2007), especially where standard regression is unavailable (Klein et al. 2014).

In a multi-state setting, the quantities of interest are the transition probabilities, $P_k(t)$ in Eq. (7). The Aalen–Johansen estimator Andersen et al. (1996) is a suitable non-parametric estimator in this context. To regress transition probabilities on features, one begins by estimating the transition probability for transition *k* at a fixed time point *t* by plugging the Aalen–Johansen estimate of Eq. (7) into Eq. (38). Then, the PV for the *k*th transition probability is given as

42 $$\begin{aligned} \widehat{\theta }_{ki}(t) = n \widehat{P_k}(t) - (n - 1) \widehat{P_k}^{-i}(t), \end{aligned}$$

which is then again used as response variable in regression models (e.g., generalized linear models or other estimating algorithms (Mogensen and Gerds 2013; Salerno and Li 2025)). One may want to compute multiple PVs at various times if the interest is a global estimate over time. As the competing risks setting is a special case of the multi-state setting, Eq. (42) still applies there, replacing the transition probabilities with an estimate of the cumulative incidence function (6), once again using the Aalen–Johansen estimator. This is illustrated in Sect. 6.2 with estimation of PV-based random forests.

#### Limitations

PVs are not applicable to left-truncated data (Grand et al. 2019) and not easily applicable to interval-censoring. For interval-censored data, the Turnbull estimator is a non-parametric estimator of the survival probability, but PVs built from this estimator do not satisfy the asymptotic properties mentioned above (Bouaziz 2023). PVs built from a parametric model for the survival function can be used (Johansen et al. 2021). The asymptotic properties of PVs hold under the assumption of fully independent censoring, which is restrictive and might not hold in practice. If censoring depends on a categorical feature, the Kaplan–Meier estimator in the PVs definition formula (39) can be replaced by a mixture of Kaplan–Meier estimators based on the different variable categories (Andersen and Pohar Perme 2010). One challenge associated with PVs is the arbitrary selection of time points at which PVs are defined. However, several sensitivity analyses have demonstrated that using 5 to 10 time points, equally spaced along the event time scale, typically provides sufficient information for reliable inference, see Andersen et al. (2003), Klein and Andersen (2005), Pohar Perme and Andersen (2008), and Andersen and Pohar Perme (2010).

#### Further reading

Recent developments in multi-state models using PVs include the extension to interval-censored data (Sabathé et al. 2020) and the relaxation of the Markov assumption (Andersen et al. 2022). PVs are also used in relative survival, where they offer an alternative that is, easier to implement in software than the existing estimator (Pavlič and Pohar Perme 2019). PVs are also useful to implement cure models (Su et al. 2022) and can be used as graphical tools to check model assumptions for hazard regression models (Cox model, additive model, Fine and Gray model), see Pohar Perme and Andersen (2008).

## Software

Several packages provide standalone implementations of specific reduction techniques for survival analysis. Notable examples include pammtools (Bender and Scheipl 2018), which provides functions for the necessary data transformations for piecewise exponential reductions, discSurv (Welchowski et al. 2022) for discrete-time data transformation, and pseudo (Maja Pohar Perme and Gerster 2017) for calculation of pseudo values in different settings. These implementations typically focus on data transformation or specialized workflows in a statistical modeling context rather than machine learning workflows.

The reduction framework described in Fig. 3 aligns naturally with the design philosophy of the mlr3pipelines package (Binder et al. 2021), which modularizes machine learning workflows into reusable and composable building blocks (PipeOps), each with well-defined train and predict semantics, standardized input/output interfaces, and internal state management. Leveraging this architecture, we implemented several reduction techniques within the mlr3proba R package (Sonabend et al. 2021) for right-censored survival data. Specifically, we provide implementations for the PEM (Sect. 3.2), DT (Sect. 3.3), and IPCW reduction (Sect. 4.1).

Each method is implemented as a self-contained pipeline consisting of two main components: a train PipeOp that performs the appropriate data transformation during training (e.g., long-format conversion for DT or PEM) and stores relevant parameters in its internal state (e.g., the cut-points or time grid), and a prediction PipeOp that maps predictions from a regression or classification model back to the survival domain using the internal state.

This design ensures full compatibility with the broader *mlr3* ecosystem, enabling flexible model selection across regression, classification, and survival learners, integrated resampling and hyperparameter tuning with nested cross-validation, and support for internal validation and early stopping (Fischer 2024), such as with XGBoost and PEM. Furthermore, transformed tasks retain the original subject identifiers, ensuring that resampling and evaluation operate on the correct observational units, rather than individual rows in the long-format data. This procedure avoids bias from pseudo-replication due to multiple rows per subject in the long-format data. This makes reductions compatible with standard performance metrics and resampling schemes in survival analysis. The pipeline design also allows high flexibility. For instance, with the PEM pipeline, users can explicitly specify the time-varying design formula (e.g., including interval end-times as features to capture time-varying effects) and can also directly control the discretization of the follow-up time.

By building on the modularity of the *mlr3* framework (Lang et al. 2019), our implementations offer a unified, extensible, and reproducible interface to reduction-based survival modeling. They enable rapid prototyping, tuning, and deployment of survival models using familiar tools from the ML ecosystem–closing the gap between modern ML methods and classical survival analysis requirements.

While the reduction pipelines implemented in mlr3proba are technically applicable to left-truncated data, competing risks and multi-state settings (i.e., data transformation and model estimation work), automated evaluation is currently not supported as this requires a refactoring of the container that stores model predictions. However, this extension will be available in future releases.

## Applications

In this section, we showcase applications of the PEM, DT, and PV reduction techniques in a single-event and a competing risks setting, using XGBoost for PEM (Chen and Guestrin 2016a) and random forests for DT and PV (Breiman 2001) as estimation algorithms. In doing so, we aim to demonstrate similarities and differences across these reduction techniques as well as their flexibility in accommodating distinct survival tasks and machine learning algorithms. While the chosen examples are deliberately simple (low-dimensional, no hyperparameter tuning, no out of sample evaluation), they illustrate that the respective approaches are in principle able to learn the underlying event time distribution, even in the presence of non-proportional hazards. A quantitative evaluation of the DT and PEM-based reductions is given in Sect. 7.2.

### Estimation of the survival function and RMST in a single-event setting

In the single-event setting, we illustrate the estimation of survival function and RMST on the tumor dataset, included in the R package pammtools (Bender and Scheipl 2018). The tumor dataset contains information on 776 patients treated for a tumor located in the stomach area. The outcome of interest is time from tumor surgery until death ($1{-}3,000$ days). In our illustrations, we consider the binary variable complications, which indicates whether or not major complications occurred during surgery.

For the PV reduction technique, the PVs were independently calculated at each time of interest and based on the non-parametric KM estimator following Eq. (39). For the single-event tumor dataset, the data transformation required for partitioning-based reduction techniques (PEM and DT) is precisely as illustrated in Sect. 3.1 (in particular, Fig. 4 and Table 2).

Figure 5 illustrates predicted survival probabilities (left panel) and the RMST (right panel) across time and stratified by complication status. Prediction curves are displayed for the DT and PEM reduction techniques (and for the Kaplan–Meier estimator as the “ground truth”), whereas for the PV approach, only point estimates are shown, underlining the fact that the PV reduction is usually used for evaluating quantities of interest at specific time points. For the PV reduction technique, we use regression random forest for the computation of predicted survival probabilities and RMST, based on Eqs. (39) and (40), respectively. We use XGBoost (Chen and Guestrin 2016a) for the PEM-based estimates (as they implement Poisson likelihood and allow inclusion of an offset) and random forest for classification for the DT approach.

Notably, all approaches, except the Cox proportional hazards model, recover the Kaplan–Meier estimates very well, although the survival curves for the two groups (complications yes vs. no) are clearly non-proportional. This means that XGBoost and RF successfully learned the interaction between the feature time and complications. The Cox proportional hazards model, stratified for the complication feature (i.e., allowing a different baseline hazard estimate for each group), will closely recover the Kaplan–Meier estimates (not shown).

While predicting survival probability curves is straightforward with the DT and PEM methods, calculating the RMST is, in general, not. Here we used the method by Zucker (1998), which becomes very complicated as more (especially continuous) features are added to the model. On the contrary, PVs are particularly useful in adjusting the estimation of restricted mean survival time conditional on features because they enable the modeling of the relation between the mean survival time and given features by a direct regression model (Andersen et al. 2004).

### Estimation of cumulative incidence

In the competing risks setting, we show the estimation of CIFs using the sir.adm dataset (Beyersmann et al. 2006). The dataset comprises a random subsample of 747 patients from the prospective SIR 3 (Spread of nosocomial Infections and Resistant pathogens) cohort study at the Charité university hospital in Berlin, Germany, which aimed to investigate the effect of hospital-acquired infections in intensive care (Wolkewitz et al. 2008). It contains information on patients’ pneumonia status at admission to the intensive care unit (ICU), time of ICU stay, and whether the patient was discharged alive or died in the hospital.

The necessary data transformation for partitioning-based reduction techniques (PEM and DT) is described in Sect. 3.1 and illustrated for an excerpt of the sir.adm dataset in Table 4. Importantly, the resulting cause-specific datasets are stacked for model estimation of partition-based reduction techniques (cf. Sect. 3.3.2). For the PV approach, survival data are transformed into PVs for each cause using the non-parametric Aalen–Johansen estimator, following Eq. (42).

**Table 4:**
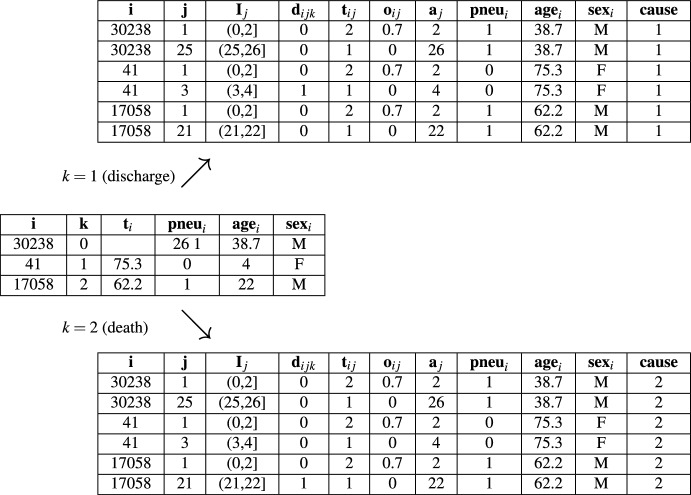
Illustration of data transformation for competing risks analysis with the sir.adm dataset

Starting from the sir.adm dataset in standard format—i.e., with a single row per individual *i* containing the ID, cause (0: censoring; 1: discharge; 2: death), observed time, and features—the follow-up time is partitioned into time intervals as illustrated in Fig. 4. Subsequently, expanded long-format datasets with one row per individual and time interval are created separately for each cause $k \in \{1,2\}$. This works analogously to Table 2, the only difference being that the event indicator is now $d_{ijk}$, indicating whether individual *i* experiences an event of type *k* in interval *j*. In addition, for each cause-specific dataset, a column denoting the cause is added

Figure 6 illustrates predicted cumulative incidence (Eq. (6)) probabilities by cause, across time, and stratified by pneumonia status. As in the single-event setting, predicted curves are displayed for the DT and PEM reduction techniques (and the non-parametric baseline Aalen–Johansen estimator); for PV, point estimates at specific time points are shown. The PVs are used as the outcome variable to estimate the cumulative incidences from a random forest regression tree.

## Empirical Investigation

The illustrative datasets contain fewer than 1,000 observations, raising the question of whether modern machine learning methods can be reliably trained without overfitting, while parametric survival models such as Weibull or log-normal AFT models remain popular for their interpretability but may degrade under model misspecification. To assess performance under controlled conditions, we first conduct a simulation study reflecting non-proportional hazards with moderate sample sizes and known covariate effects in Sect. 7.1. In Sect. 7.2, we then complement this with a benchmark experiment on real and synthetic datasets, comparing PEM and DT reduction techniques to common survival learners using the *mlr3* framework (Sect. 5), focusing on approaches that estimate the event time distribution.

For both empirical investigations, preprocessing was applied as part of the learner pipeline, which included imputing missing factor levels during prediction (if any), combining rare factor levels in high-cardinality factor variables, and removing constant variables if any were introduced in previous steps, all of which are common techniques applied for computational robustness (Thomas 2024).

In setting up the tuning procedure, we closely follow Burk et al. (2024): we employ a nested resampling tuning procedure (Bischl et al. 2023) using 3-fold cross-validation for inner resampling and repeated 3-fold cross-validation with a variable number of repetitions: three repetitions for tasks with fewer events ($\le 500$ events), one repetition for tasks with over 1000 events, and 2 repetitions otherwise. Hyperparameters are tuned via Bayesian optimization (Garnett 2023), using a hybrid tuning budget of either a set number of evaluations scaling with the number of tunable parameters per learner ($50 \cdot n_\theta$) or until a time limit of 120 h (five days) was reached. XGBoost uses early stopping for the nrounds parameter (stopping after 50 iterations without improvement).

The experiments use the C-Index for evaluation and the integrated survival brier score (ISBS; Graf et al. 1999) for tuning and evaluation, where it integrates up to the 80th percentile of observed times in each training set (Sonabend et al. 2024).

### Simulation Study

To assess the robustness of model performance to sample size, we simulate survival data mirroring the illustrative example, i.e., reflecting non-proportional hazards. Each replication generates samples of size 750 (50 replications) with two informative covariates—a continuous variable $x_1 \sim \mathscr {N}(0,1)$ and a binary variable $x_2 \sim \mathscr {B}(0.5)$ (e.g., treatment)—as well as an additional noise variable $x_3 \sim \mathscr {U}(-1,1)$; the latter one was included in the feature space of all machine learning methods. Event times follow a Weibull distribution with a shape parameter depending on $x_2$ ($\alpha = 1$ if $x_2 = 1$, otherwise $\alpha = 3$), inducing non-proportional hazards. The scale parameter is governed by the log-hazard-level linear predictor $\eta = -3.1 + 1.0 x_1 + 0.9 x_2-0.3 x_1 x_2$, allowing both main and interaction effects. We impose approximately 20% random censoring generated from an exponential distribution (by setting the rate $\lambda = 0.07$) and an additional 10% administrative censoring after time 10 to mimic realistic survival data settings.

As a non-parametric baseline, we include the Kaplan–Meier estimator (KM). To mirror the data-generating process, we employ a Weibull accelerated failure time (AFT) non-proportional hazards (NPH) model with an interaction term (AFT_WB_NPH). Additional misspecified parametric AFT models include a Weibull AFT NPH without interaction (AFT_WB_NPH_NINT), a Weibull AFT PH model with interaction (AFT_WB_PH), a log-normal AFT model with and without interaction (AFT_LN_NPH, AFT_LN_NPH_NINT) and a log-normal AFT PH model without the interaction term (AFT_LN_PH_NINT). All parametric models are implemented via standard parametric survival models using flexsurvreg and flexible spline models using flexsurvspline from the *flexsurv* R package Jackson (2016). For semi-parametric modeling, we include the Cox proportional hazards model with (CPH) and without (CPH_NINT) interaction. As machine-learning-based approaches, we consider the random survival forest (RSFRC) and XGBoost combined with the piecewise exponential reduction framework. For the latter, we consider two variants: one that offers full flexibility with interactions (XGB_PEM) and one that restricts interactions (XGB_PEM_NINT) (Chen and Guestrin 2016b).

**Fig. 7:**
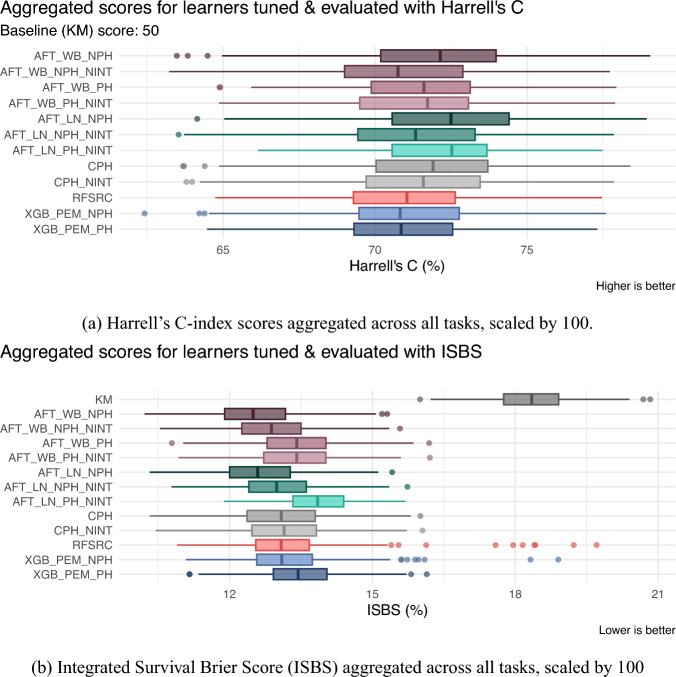
Aggregated simulation study results for 50 datasets with $n=750$ presented as boxplots, where AFT_WB_NPH is the correctly specified model

Results are presented aggregated per learner across all datasets with $n=750$ in Fig. 7. The results indicate that even on small sample sizes, the reduction-based approach has competitive performance to survival-specific ML learners (XGB_PEM vs. RFSRC). Both learners perform better than the misspecified Weibull models as well as the CPH models (that are both misspecified because of the proportional hazards assumption and CPH_NINT additionally due to lack of interaction effect). In general, models that do not allow for different shapes of the baseline hazard depending on $x_2$ perform worst. Misspecification w.r.t. missing the interaction effect on the other hand doesn’t seem to affect the results too much (probably because the interaction effect was small in this setup, Pencina and D’ Agostino Sr RB, D’ Agostino Jr RB, Vasan RS 2008; Harrell and Frank 2015; Austin 2017). The log-normal model performs very well in this setting when the parameters of the model are correctly specified, although the model is generally misspecified as it assumes a wrong distribution of event times. In this setting, however, this doesn’t seem to matter too much, because the log-normal in this case can approximate the baseline hazard imposed by the Weibull distribution. Notably, neither RFSRC nor XGB_PEM require any information about the true data generating process and are able to learn the distribution of event times. For ablation, we additionally fit an alternative XGB PEM model where we forced the hazards to be proportional (XGB_PEM_PH), which clearly degrades the performance.

### Benchmarking experiments

The experiment comprises nine tasks with seven real-world datasets available in R packages from CRAN and two synthetic datasets simulated to illustrate specific challenges in predictive modeling for survival tasks (see Table 5). synthetic-breakpoint simulated data with a change point in the baseline hazard. synthetic-tve contains simulated data with highly non-linear time-varying effects (i.e. non-proportional hazards). For the general setup of the benchmark, including choice of hyperparameter space, we followed the large scale benchmark in Burk et al. (2024). The goal of the benchmark in this paper was not to perform an exhaustive large-scale comparison of different learners, but rather to illustrate that predictive performance of reduction techniques is largely on par with survival-specific learners.

For comparison, we evaluate learners available via the *mlr3extralearners* R package (Fischer et al. 2025), including Kaplan–Meier (KM) as a baseline, $L_2$-penalized Cox regression (RIDGE), the Cox elastic net (GLMN), random survival forest (RSFRC) and classification random forest with discrete-time reduction (RSFRC_DT). In addition, we consider three XGBoost variants: one with a Cox-based objective (XGBCox), a piecewise exponential model reduction (XGB_PEM), and a discrete-time reduction (XGB_DT).

Dedicated factor encoding was applied for GLMN and RIDGE, as they are the only learners which do not natively handle factors. Their underlying implementation also performs scaling of features automatically, which is not required for the tree-based methods due to their invariance to linear transformations.

The experiment is conducted twice in total, once using Harrell’s C-index for tuning and evaluation (Harrell et al. 1982), and once using the integrated survival brier score (ISBS; Graf et al. 1999) analogously, where we integrate up to the 80th percentile of observed times in each training set (Sonabend et al. 2024). To ensure consistency and fairness of our results, we employ the KM estimator as a fallback learner (e.g. Fischer et al. 2024) which is used to impute performance scores in cases where the learner in question can not produce a result due to underlying errors, such as numerical issues or other implementation-specific reasons; we observed that occasional failures of XGBoostCox arise from implementation-level instabilities that produce undefined evaluation metrics (NaN). This also results in learners with many errors producing results skewed towards the performance of the baseline KM results, making stability an indirect additional evaluation metric.

**Fig. 8:**
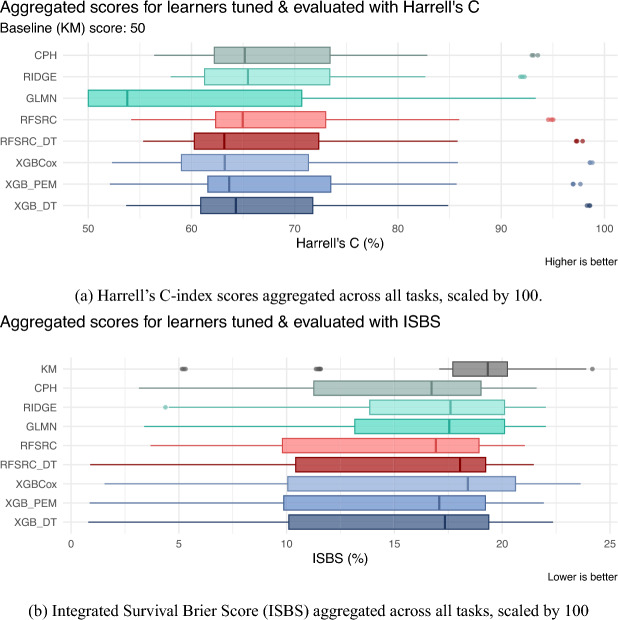
Aggregated benchmark results across all tasks presented as boxplots

**Fig. 9:**
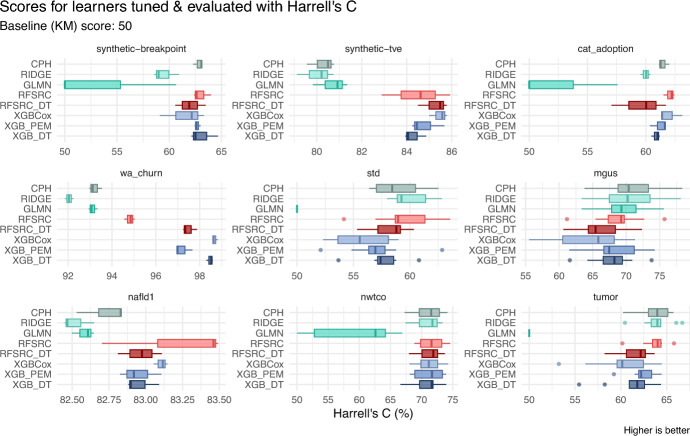
Boxplots of Harrell’s C evaluation scores across each task’s outer resampling iterations, scaled by 100. Baseline (KM) scores are omitted as they are equal to 50 in all cases

**Fig. 10:**
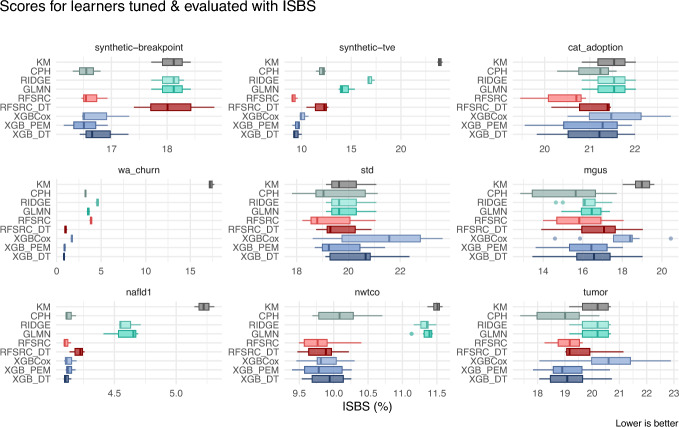
Boxplots of integrated survival Brier scores (ISBS) scores across each task’s outer resampling iterations, scaled by 100

Results are presented aggregated per learner across all tasks in Fig. 8, and separately for each task aggregated across outer resampling iterations in Figs. 9 and 10 in Appendix A, where we also include tables of evaluation scores. The results indicate that overall the reduction based approaches have competitive performance to survival-specific learners. However, the performance appears to depend to some extent on the underlying classification/regression learner. For example, while XGBoost based DT reduction performs quite well, while the random forest based DT reduction underperforms on many tasks compared to random survival forest. On the other hand, both PEM and DT reductions with XGBoost outperform the XGBoost based Cox implementation, which frequently errored during estimation and thus appears to be less stable compared to the respective reductions (in its current implementation). Further, results show a considerably longer runtime of reduction-based techniques for larger datasets. For example, on average across outer resampling iterations, RFSRC_DT has a runtime of 1540 min compared to 180 for RFSRC. This is because the implemented pipeline uses observed event times as cut points, so the size of the transformed dataset grows with the number of unique event times. Similarly, Kvamme and Borgan (2021) show that finer time discretization increases model complexity in discrete-time neural networks. However, they further suggest that a small number of well-chosen cut points can be sufficient.

## Discussion

In this work, we proposed a general strategy for reducing complex survival analysis tasks to standard regression or classification tasks—which in turn enables the application of standard machine and deep learning methods—and provided an overview of such reduction techniques. Overall, these reduction techniques account for the particular incompleteness of information in survival data (e.g., right-censoring and left-truncation), are applicable to a broad range of survival tasks (e.g., competing risks or multi-state settings), can predict different quantities of interest (e.g., hazards, CIFs), and can use off-the-shelf implementations of machine or deep learning methods (e.g., boosting, random forest), while not imposing (strong) assumptions regarding the underlying distribution of event times. Our implementation in mlr3proba provides a principled way to make such reductions available for practitioners. Beyond the specific implementation, we provide a blueprint for the implementation of such techniques in general ML workflows.

Empirical results from selected data examples, a simulation study on non-proportional hazards, and a benchmark study on multiple real-world and synthetic datasets indicate that standard machine learning algorithms for regression and classification in combination with reduction techniques are competitive with established survival-specific learners.

Despite their general applicability, each reduction technique also comes with its own limitations. For instance, PEMs and DT models are not applicable to left-censored data, while PV-based approaches are not applicable to left-truncated data. None of the techniques can easily incorporate individual-specific interval-censored data. Practitioners should be aware that reduction-based methods can exhibit longer runtimes on larger datasets, as using all event times as cut points inflates the transformed dataset and computational cost; selecting a smaller set of well-chosen cut points may help mitigate this trade-off. At the same time, while our current benchmark experiments were comprehensive, they could be more exhaustive regarding hyperparameter tuning and choice of learners. Currently, the implementation of reduction techniques within mlr3proba is limited to PEM, DT and IPCW approaches, with automated evaluation not yet supported for many survival tasks beyond single-event, right-censoring data.

Future work will look at other reduction techniques as well as at extensions of the existing ones to survival settings currently not covered. In particular, PV based reductions will be integrated in mlr3proba. Moreover, further empirical evaluation of different base learner/reduction technique combinations could elicit a better understanding of the underlying processes and reasons for differences in performance.

## Figures and Tables

**Fig. 11:**
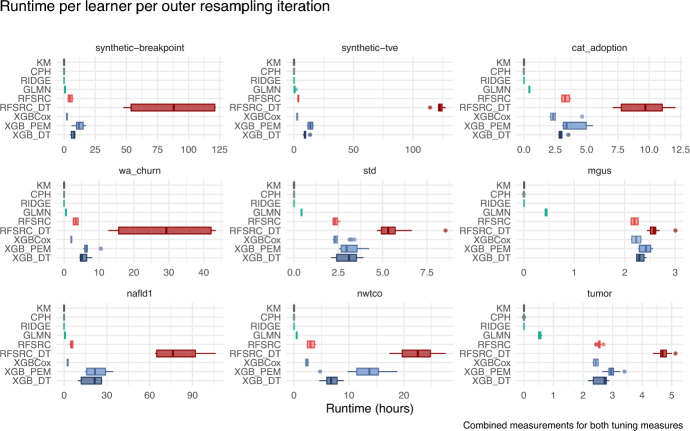
Boxplots of learner runtime (hours) per task across resampling iterations, which includes tuning and final model fitting

## Data Availability

All datasets used in this work (Sects. 6, 7.1, and 7.2) are public datasets, accessible via the anonymous GitHub repositories. - https://anonymous.4open.science/r/reduction-techniques-F517, - https://anonymous.4open.science/r/reduction-techniques-benchmark-4FB7

## Appendix A

### Additional benchmark details and results

See Tables 5, 6, 7, 8 and 9.

**Table 5:** Tasks used in benchmark comparison including number of observations (N), features (p), and observed events, the censoring rate, and number of repeats for outer repeated cross-validation used for evaluation

Task	N	p	Events	Cens. %	Repeats
cat_adoption	2257	18	1434	36.46	1
mgus	176	7	165	6.25	3
nafld1	12,588	5	1018	91.91	1
nwtco	4028	3	571	85.82	2
std	877	21	347	60.43	3
synthetic-breakpoint	2000	20	1108	44.60	1
synthetic-tve	2000	24	1508	24.60	1
tumor	776	7	375	51.68	3
wa_churn	7032	18	1869	73.42	1

**Table 6:** Learners used in the benchmark comparison, including their *mlr3* IDs, implementing R packages, and the number of explicitly tuned hyperparameters

ID	Package	mlr3 ID	# Parameters
KM	survival	surv.kaplan	0
CPH	survival	surv.coxph	0
RIDGE	glmnet	surv.cv_glmnet	0
GLMN	glmnet	surv.cv_glmnet	1
RFSRC	randomForestSRC	surv.rfsrc	5
RFSRC_DT	randomForestSRC	classif.rfsrc	5
XGBCox	xgboost	surv.xgboost.cox	5
XGB_PEM	xgboost	regr.xgboost	5
XGB_DT	xgboost	classif.xgboost	5

cv.glmnet internally tunes the lambda regularization parameter

See Figs. 9, 10, and 11.

**Table 7:** Mean (SD) of evaluation scores aggregated by learner

Learner	Harrell’s C	ISBS
KM	50 (0)	17.87 (4.56)
CPH	68.87 (9.73)	14.93 (5.52)
RIDGE	68.67 (9.51)	16.04 (5.3)
GLMN	61.89 (14.12)	15.86 (5.45)
RFSRC	69.11 (10.28)	14.78 (5.52)
RFSRC_DT	67.98 (11.36)	15.23 (6.04)
XGBCox	67.12 (12.31)	15.81 (6.51)
XGB_PEM	68.4 (11.31)	14.79 (6.04)
XGB_DT	68.19 (11.44)	14.99 (6.18)

Scores scaled by 100 for readability

**Table 8:** Mean (SD) of evaluation scores for each learner and task

Learner	Harrell’s C	ISBS
*synthetic-breakpoint*		
KM	50 (0)	18.09 (0.36)
CPH	62.82 (0.47)	16.55 (0.25)
RIDGE	59.5 (1.27)	18.05 (0.3)
GLMN	53.55 (6.15)	18.09 (0.36)
RFSRC	63 (0.88)	16.64 (0.25)
RFSRC_DT	62 (1.45)	18.09 (0.73)
XGBCox	61.53 (2.17)	16.77 (0.48)
XGB_PEM	62.68 (0.29)	16.53 (0.39)
XGB_DT	63.1 (1.37)	16.8 (0.45)
*synthetic-tve*		
KM	50 (0)	23.91 (0.26)
CPH	80.28 (0.63)	12.01 (0.51)
RIDGE	80.02 (0.83)	16.92 (0.38)
GLMN	80.7 (0.79)	14.39 (0.85)
RFSRC	84.47 (1.53)	9.25 (0.31)
RFSRC_DT	85.25 (0.67)	11.82 (1.17)
XGBCox	85.46 (0.41)	10.15 (0.47)
XGB_PEM	84.79 (0.77)	9.55 (0.41)
XGB_DT	84.28 (0.5)	9.5 (0.46)
*cat_adoption*		
KM	50 (0)	21.46 (0.6)
CPH	61.46 (0.48)	21.04 (0.68)
RIDGE	59.98 (0.43)	21.46 (0.6)
GLMN	52.52 (4.37)	21.46 (0.6)
RFSRC	62.05 (0.48)	20.36 (0.79)
RFSRC_DT	59.6 (2.36)	21.01 (0.74)
XGBCox	61.94 (1.02)	21.59 (1.15)
XGB_PEM	61.21 (0.74)	20.93 (1.23)
XGB_DT	60.89 (0.37)	21.02 (1.1)
*wa_churn*		
KM	50 (0)	17.32 (0.32)
CPH	93.21 (0.3)	3.23 (0.08)
RIDGE	92.05 (0.2)	4.56 (0.16)
GLMN	93.12 (0.2)	3.56 (0.17)
RFSRC	94.81 (0.24)	3.84 (0.14)
RFSRC_DT	97.49 (0.34)	1.01 (0.14)
XGBCox	98.68 (0.13)	1.7 (0.14)
XGB_PEM	97.2 (0.41)	0.92 (0.09)
XGB_DT	98.48 (0.15)	0.86 (0.08)
*std*		
KM	50 (0)	19.92 (0.71)
CPH	58.94 (2.18)	19.59 (1.23)
RIDGE	60.13 (1.73)	19.92 (0.71)
GLMN	50 (0)	19.92 (0.71)
RFSRC	59.36 (2.79)	19.29 (0.99)
RFSRC_DT	58.19 (1.84)	19.69 (0.78)
XGBCox	55.74 (2.63)	21.29 (1.86)
XGB_PEM	57.11 (2.9)	19.71 (1.09)
XGB_DT	57.58 (1.9)	20.37 (1.04)
*mgus*		
KM	50 (0)	19.01 (0.52)
CPH	70.3 (4.55)	15.42 (1.83)
RIDGE	70.18 (4.65)	16.15 (0.9)
GLMN	69.65 (3.58)	16.34 (0.81)
RFSRC	68.71 (4.14)	15.87 (1.41)
RFSRC_DT	66.3 (4)	16.65 (1.77)
XGBCox	64.49 (5.22)	17.88 (1.72)
XGB_PEM	68.3 (4.71)	16.11 (1.57)
XGB_DT	67.83 (3.59)	16.36 (1.69)
*nafld1*		
KM	50 (0)	5.23 (0.08)
CPH	82.73 (0.17)	4.14 (0.05)
RIDGE	82.52 (0.11)	4.6 (0.1)
GLMN	82.58 (0.08)	4.59 (0.15)
RFSRC	83.22 (0.45)	4.11 (0.03)
RFSRC_DT	82.96 (0.15)	4.21 (0.06)
XGBCox	83.11 (0.05)	4.13 (0.05)
XGB_PEM	82.95 (0.14)	4.14 (0.04)
XGB_DT	82.96 (0.12)	4.11 (0.03)
*nwtco*		
KM	50 (0)	11.49 (0.08)
CPH	71.13 (2.57)	10.1 (0.39)
RIDGE	70.99 (2.28)	11.33 (0.11)
GLMN	59.39 (7.5)	11.35 (0.12)
RFSRC	71.6 (2.3)	9.81 (0.33)
RFSRC_DT	71.29 (2.13)	9.84 (0.28)
XGBCox	71.18 (2.24)	9.87 (0.29)
XGB_PEM	71.2 (2.62)	9.83 (0.35)
XGB_DT	70.79 (2.58)	9.91 (0.3)
*tumor*		
KM	50 (0)	20.06 (0.56)
CPH	63.79 (1.69)	18.91 (0.98)
RIDGE	63.9 (1.84)	20.06 (0.56)
GLMN	50 (0)	20.06 (0.56)
RFSRC	63.61 (1.57)	19.07 (0.5)
RFSRC_DT	61.42 (1.89)	19.63 (0.73)
XGBCox	60.05 (3.58)	20.43 (1.44)
XGB_PEM	62.33 (1.52)	19.16 (0.93)
XGB_DT	61.17 (2.79)	19.2 (0.85)

Scores scaled by 100 for readability

**Table 9:** Runtime (mean (SD), minutes) and memory usage (mean (sd), MB) per learner across outer resampling iterations

Learner	Memory (MB)	Runtime (Minutes)
KM	213.3 (10.3)	0.3 (0.1)
CPH	213.8 (10.4)	0.1 (0)
RIDGE	233.9 (10.3)	0.3 (0.1)
GLMN	260.5 (11)	30 (7.7)
RFSRC	303.9 (61.6)	180 (66.9)
RFSRC_DT	1849.1 (2271.5)	1540.1 (2243.6)
XGBCox	261.3 (11.1)	143.9 (21.5)
XGB_PEM	251.5 (7.3)	418.3 (391.3)
XGB_DT	251.6 (7.5)	308.4 (286.8)

This includes tuning (if applicable) and final model fitting

## Code availability

All code required for running the application, simulation, and benchmark analyses in this work (Sects. 6, 7.1, and 7.2), as well as for creating the corresponding figures, is available from the anonymous GitHub repositories - https://anonymous.4open.science/r/reduction-techniques-F517 - https://anonymous.4open.science/r/reduction-techniques-benchmark-4FB7.
